# Supplementary material for: Hydrogen and dark oxygen drive microbial productivity in diverse groundwater ecosystems
Source: Nat Commun. 2023 Jun 13;14:3194. doi: 10.1038/s41467-023-38523-4 (PMC10264387; doi:10.1038/s41467-023-38523-4)
Supplement: Supplementary file 1 — Supplementary Information [file 41467_2023_38523_MOESM1_ESM.pdf]

Supplementing Information for

Hydrogen and dark oxygen drive microbial productivity in diverse groundwater ecosystems

Author list

S. Emil Ruff[1,2,3*], Pauline Humez[1], Isabella Hrabe de Angelis[1,4], Muhe Diao[1], Michael Nightingale[1], Sara Cho[1], Liam Connors[1], Olukayode O. Kuloyo[1], Alan Seltzer[5], Samuel Bowman[5], Scott D. Wankel[5], Cynthia N. McClain[1,6,7], Bernhard Mayer[1], Marc Strous[1]

Affiliations

[1] Department of Geoscience, University of Calgary, Calgary, Canada.

[2] Josephine Bay Paul Center, Marine Biological Laboratory, Woods Hole, USA.

[3] Ecosystems Center, Marine Biological Laboratory, Woods Hole, USA.

[4] Multiphase Chemistry Department, Max Planck Institute for Chemistry, Mainz, Germany.

[5] Department of Marine Chemistry and Geochemistry, Woods Hole Oceanographic Institution, Woods Hole, USA.

[6] Alberta Environment and Protected Areas, Calgary, Canada.

[*] Corresponding author: eruff@mbl.edu, ORCID: 0000-0002-6872-6188

[7] Alberta Biodiversity Monitoring Institute, Edmonton, Canada

This PDF file includes:

Supplementary Text
Supplementary Figures 1 to S15
Supplementary Table S1

Supplementary Results

Further results on section: Geochemical evolution of groundwater in the Canadian Prairie

Geologic formations

Situated in the Western Canadian Sedimentary Basin, Alberta's conventional and unconventional hydrocarbon reserves include bitumen in northern Alberta (i.e. Athabasca oil sands deposits in Fort McMurray, Cold Lake and Peace River), shale gas and tight gas in the northwest (i.e. Duvernay shale, Montney tight sandstone/shale, Muskwa formation), and coal bed methane (CBM) in south-central Alberta (i.e. coal zones from the Horseshoe Canyon Formation, Belly Group, Mannville Group)[1] (Fig. 1). The terrestrial fluvial Paskapoo Formation consists of mudstone and siltstone, with thick tabular sandstones deposited in fluvial channels[2–4]. The terrestrial fluvial Horseshoe Canyon Formation consists primarily of sandstone, siltstone, and coal. The marine Bearpaw formation interfingers with the lower and middle Horseshoe Canyon formation and consists predominantly of shale and minor sandstone and coal[3]. The Belly River Group consists of fluvial sandstone and siltstone with minor mudstone and coal. The coal zones with coal bed methane (CBM) potential are included in the Horseshoe Canyon and the Belly River formations. Thin coal seams may also occur in the Paskapoo Formation. All these bedrock formations comprise heavily used aquifers in the prairies region of Alberta[5] although they are highly heterogeneous. Neogene-Quaternary surficial deposits of varying thickness and composition (e.g., till) overlie bedrock and are associated with glacial retreat. Glacial sand and gravel deposits that fill buried river valleys (channels) are also commonly used aquifers in Alberta (Supplementary Table 1). Consequently, the GOWN wells (Groundwater Observation Well Network) are completed in many different lithologies including sandstone, siltstone with intermittent coal or shale beds, pre-glacial sand, and sandy and gravelly lacustrine or moraine deposits. More information about these geological formations can be found in[2–9].

Supplementary Table 1: Depositional environments and associated sediments of the groundwater monitoring wells investigated in this study.

**Depositional Environments**		**# of wells**	**Formation**
Neogene-Quaternary surficial deposits			
Buried Channel*<br>(V: valley; a: preglacial fluvial incision)		16	Medicine Hat V[a], Lethbridge V[a], Keho V, Irvine V, Sibbald V, Vermilion V, Bronson V
Undifferentiated/Glacial tills		22	
Upper Cretaceous to Paleocene			
Coarse sandy clastic deposits<br>(terrestrial environment)		3<br>7<br>4<br>9	Milk River,<br>Belly River Group (e.g., Oldman),<br>Horseshoe Canyon,<br>Scollard/Paskapoo,
Organic-rich/coal seams<br>(terrestrial environment)		1<br>6<br>2	Belly River,<br>Horseshoe Canyon,<br>Paskapoo
Fined-grained deposits<br>*(shale, siltstone, mudstone)*	Marine environment /transgression	4<br>2	Bearpaw,<br>Loon River
	Terrestrial environment	1<br>3<br>14	Belly River Group (e.g., Bulwark, Foremost),<br>Horseshoe Canyon,<br>Scollard/Paskapoo
	Undifferentiated	1	
**Buried valleys are referring to valleys that are incised into bedrock and are buried by till.*			

Hydrochemical facies

Hydrochemical facies are defined as groundwater masses that have different geochemical attributes[10]. These hydrochemical facies are frequently compared using graphical representation[11]. The traditional Piper diagram in Figure 2a indicates that there are different hydrochemical facies within the groundwater samples varying from Ca-Mg-HCO_3 to Na-HCO_3, SO_4-rich water as well as mixing water facies. Access to groundwater data using radioisotopes such as tritium and $^{14}C_{DIC}$ permits to estimate the travel time between the point of recharge and the point of sampling[12]. The chemical evolution using the Ca/Na mass ratios proxy and preliminary age dating data permit to delineate further those hydrochemical facies (Figure 2b). In this study, the youngest tritium-containing groundwater samples (indicating recharge after 1960) were characterized by low total dissolved solids (< 400 mg L^{-1}), high Ca/Na ratio (median of 3.5) and a calcium-magnesium-bicarbonate dominated water chemistry resulting from dissolution of carbonates during recharge (yellow symbols, Fig. 2). Those groundwater samples were collected from wells completed mainly in Neogene-Quaternary surficial deposits. In contrast, the most evolved groundwater samples containing no tritium and having $^{14}C_{DIC}$ ages (uncorrected) indicative of groundwater more than several hundreds of years old, had elevated average contents of total dissolved solids (>900 mg L^{-1}) and a low Ca/Na ratio (median of 0.01). Old waters were rich in sodium, bicarbonate, and chloride due to water-rock interactions including ion exchange, and weathering of minerals (purple symbols, Fig. 2). Those groundwater samples were collected from wells completed mainly in buried valleys, Paskapoo, Horseshoe Canyon and Milk River formations. Sulfate derived from oxidation of reduced-S, anhydrite or gypsum dissolution is ubiquitous in a third group of groundwater samples characterized by variable groundwater ages, elevated total dissolved solids (> 1700 mg L^{-1}) and low Ca/Na (median of 0.12) (blue symbols, Fig. 2). These sulfate-rich waters are mainly associated with sodium-sulfate, sodium-bicarbonate-sulfate hydrochemical facies (low Ca/Na). A minority of samples is associated with calcium-sodium-bicarbonate-sulfate hydrochemical facies resulting in higher Ca/Na ratios. Those groundwater samples were collected from wells completed in Neogene-Quaternary surficial deposits or in the Cretaceous Paskapoo, Horseshoes Canyon, Bearpaw, Loon River formations. The groundwater mixture often results in intermediate hydrochemical facies (red symbols, Fig. 2) associated with calcium-sodium-bicarbonate-chloride waters. These groundwater samples are collected from wells mainly completed in Neogene-Quaternary surficial deposits.

^{14}C-based Groundwater dating

Reimer and colleagues[13] point out the problems with the different conventions in radiocarbon measurements for post-bomb ^{14}C data and define a fractionation-corrected fraction modern (the $F^{14}C$ value) according to the amended conventions[14]:

$$F^{14}C = \frac{^{14}A_{SN}}{^{14}A_{ON}} \quad (1)$$

where $^{14}A_{SN}$ and $^{14}A_{ON}$ refer to the fractionation-corrected or normalized ^{14}C activity for the sample and the secondary Oxalic acid-II (Ox2) standard used for the ^{14}C-measurements[15] respectively. Both specific activities of the sample and Ox2 $^{14}A_S$ and $^{14}A_{ox2}$ are first measured and then normalized to $\partial^{13}C$= -25 ‰ giving the normalized sample activities $^{14}A_{SN}$ and $^{14}A_{ON}$ as follow:

$$^{14}A_{SN} = \ ^{14}A_S * \left(\frac{1+\frac{^{13}\delta(-25)}{1000}}{1+\frac{^{13}\delta(sample)}{1000}}\right)^2 = \ ^{14}A_S * \left(\frac{0.975}{1+\frac{^{13}\delta(sample)}{1000}}\right)^2 \quad (2)$$

For the standard according to Mook and van der Plicht[16]

$$^{14}A_{ON} = 0.95 * \ ^{14}A_{ox1} * \left(\frac{1+\frac{^{13}\delta(-19)}{1000}}{1+\frac{^{13}\delta(Ox1)}{1000}}\right)^2 = 0.7459 * \ ^{14}A_{ox2} * \left(\frac{1+\frac{^{13}\delta(-25)}{1000}}{1+\frac{^{13}\delta(Ox2)}{1000}}\right)^2 = 0.7459 * \ ^{14}A_{ox2} * \left(\frac{0.975}{1+\frac{^{13}\delta(Ox2)}{1000}}\right)^2 \quad (3)$$

Thus, from equations (1), (2) and (3):

$$\mathrm{F}^{14}\mathrm{C} = \frac{^{14}A_S}{0.7459 * ^{14}A_{ox2}} * \left(\frac{1+\frac{^{13}\delta(Ox2)}{1000}}{1+\frac{^{13}\delta(sample)}{1000}}\right)^2 \quad (4)$$

$F^{14}C$ can thus ranges between 0 and 1 (1 for modern value) or 0 to 100 % modern carbon.

The conventional radiocarbon age is given by:

$$Age = -8033 * \ln(F^{14}C) \quad (5)$$

with the half-life value $T_{1/2}$ of 5568 years divided by ln(2) resulting in the value 8033. The ^{14}C ages thus calculated are reported in BP (Before Present). The term Present does not correspond to the present day but it refers to the standard activity 1950 AD. The measured activity plus error $F^{14}C \pm \sigma(F^{14}C)$ reported can be translated into an age T± σ(T).

Younger, recently recharged groundwater

A total of 17 % (20 out of 112) groundwater samples fell in the Ca^{2+}-Mg^{2+}-HCO_3^- (calcium-magnesium-bicarbonate) hydrogeochemical facies (yellow symbols; Fig. 2a). The water has on average a low concentration of Total Dissolved Solids (TDS) of 361 ± 125 mg L^{-1} (n=20). Among the alkaline earths, the concentration of Ca^{2+} and Mg^{2+} ions ranged from 26.8 to 142 mg/L and 13.0 to 52.9 mg L^{-1} with a mean of 77.8 ± 29.9 mg L^{-1} and 27.1 ± 10.2 mg L^{-1} (n=20) respectively. Sodium concentrations were on average 24.7 ± 8.8 mg L^{-1}. Such facies are marked by a mass ratio $Ca^{2+}/Na^+ > 0.7$ (Fig. 2b). For the anions, the average concentrations of HCO_3^-, SO_4^{2-}, NO_3^-, Cl^- are 34.5 ± 77.3 mg L^{-1}, 37.5 ± 42.2 mg L^{-1}, 10.6 ± 26.7 mg L^{-1}, 10.5 ± 21.9 mg L^{-1} (n=20) respectively. Values of dissolved inorganic carbon $\delta^{13}C_{DIC}$ ranged from -12 ‰ to -16 ‰ suggesting carbonate minerals dissolution in closed and open systems, respectively. Environmental tracers such as ^{14}C, ^3H have helped refine estimates of recharge, flow time scales and groundwater age. Groundwater age refers to the travel time between the point of recharge and the point of sampling. A subset of nine samples has been submitted for ^3H and $^{14}C_{DIC}$ analyses. The relatively high ^3H content (mean of 9.5 Tritium Units (T.U.), n=7) indicates that these waters had a relatively short residence time (post-year 1953). Only two out of nine younger waters had ^3H values below detection limit <0.8 T.U. $F^{14}C_{DIC}$ had an average of 68.2 ± 15.3 (n=13) with fractions varying from 40 to 84 % modern carbon, similar to previously characterized young groundwater of 85±5 % modern carbon[12].

Groundwater of intermediate age

A total of 10 % (11 of 112) groundwater samples fell into mixed groundwater hydrochemical facies dominated by Na^+, Ca^{2+} and/or Mg^{2+} and HCO_3^- and/or Cl^- (red symbol, Fig. 2a). The water had an average TDS concentration of 572 ± 243 mg L^{-1} (n=11). The concentrations of Na^+, Ca^{2+} and Mg^{2+} were on average 87.2 ± 27.9 mg L^{-1}, 72.8 ± 31.5 mg L^{-1}, 44.1 ± 36.1 mg L^{-1} respectively, with mass ratios of 0.3 < Ca^{2+}/Na^+ <1.1 (Fig. 2b). HCO_3^- content was 483 ± 196 mg L^{-1}, SO_4^{2-} was 66.3 ± 70.3 mg L^{-1}, Cl^- was 56.6 ± 120 mg L^{-1}, and NO_3^- was only detected in 4 samples. When detected, the concentration of NO_3^- was 0.13 mg L^{-1}. A subset of 7 groundwater samples out 12 were submitted for ^3H and $^{14}C_{DIC}$ analyses. While the $F^{14}C_{DIC}$ values show a wide range from <0.5 (detection limit) to 80 % modern carbon, few groundwater samples are associated with ^3H > 0.8 TU (n =2, up to 11.7 TU).

Older, geochemically mature groundwater

A total of 51 % (57 of 112) groundwater samples fell into the Na^+-HCO_3^--Cl^- (sodium-bicarbonate-chloride) hydrochemical facies (purple symbols; Fig. 2a). The water had an average TDS concentration of 1103 ± 889 mg L^{-1} (n=57). Sodium was the dominant cation with an average concentration of 427 ± 359 mg L^{-1}. A significant decrease in the concentration of Ca^{2+} was observed with an average of 12.8 ± 21.3 mg L^{-1}. The mass ratio Ca^{2+}/Na^+ was <0.5 (Fig. 2b). Bicarbonate concentration was 664 ± 343 mg L^{-1} (n=112), Cl^- was on average 67 ± 126 mg L^{-1}, with 7 outlier samples featuring concentrations of Cl^- > 500 mg L^{-1}. Sulfate was in average < 131 mg L^{-1}, nitrate was not detected except in two samples containing nitrate concentrations of > 3 mg L^{-1}. A subset of 30 groundwater samples was submitted to ^3H and $^{14}C_{DIC}$ analyses. The ^3H content of 90 % of old groundwater samples was below the detection limit, only three samples showed a ^3H > 0.8 TU. The ^{14}C activity of 8 old groundwater samples was below the detection limit of the radiocarbon technique, while 22 samples had an average $F^{14}C_{DIC}$ of 17.8 ± 17.7 modern carbon.

Older, sulfate-rich groundwater

A total of 21 % (24 of 112) groundwater samples fell into hydrochemical facies dominated by SO_4^{2-} (blue symbols; Fig. 2a). These waters had an average TDS concentration of 2882 ± 3555 mg L^{-1} directly correlated with average SO_4^{2-} concentrations of 1563 ± 2423 mg L^{-1}. The average content of HCO_3^- is 650 ± 287 mg L^{-1}. The major cation concentrations varied and allowed to identify two sub-categories of sulfate-rich waters, those dominated by Ca^{2+} and those dominated by Na^+. In the former, the calcium concentration was 139 ± 79.2 mg L^{-1} (n=4), magnesium was 54.4 ± 30.1 and sodium was 92.2 ± 90.0 mg L^{-1}. Resulting in a mass ratio of Ca^{2+}/Na^+ > 1 (Fig. 2b). The hydrochemical type of these waters develops into Ca^{2+}-Mg^{2+}-HCO_3^--SO_4^{2-}. In the Na^+-rich waters, the sodium concentration was 870 ± 915 mg L^{-1} (n=20), calcium was 93.8 ± 130 mg L^{-1} (n=20), and magnesium was 85.8 ± 183. This results in a mass ratio of Ca^{2+}/Na^+ <0.8. The hydrochemical type of the water develops into Na^+-(HCO_3^-)-SO_4^{2-}. A subset of 8 groundwater samples was submitted for ^3H and $^{14}C_{DIC}$ analyses. While the $F^{14}C_{DIC}$ values showed a wide range from <0.5 (detection limit) to 80 % modern carbon, few groundwater samples were associated with ^3H > 0.8 TU (up to 8.3 TU). For both subtypes, the $\delta^{13}C_{DIC}$ values had a narrower range between -20 ‰ to -12 ‰ indicating that most of the DIC content resulted from organic matter oxidation and/or carbonate mineral interactions in these systems.

Methane and higher alkanes

Methane was present in all groundwater samples in a wide range of concentrations from 0.006 to >74 mg L^{-1}. The mean and median concentrations of methane were 6.87 ± 15.9 mg L^{-1} and 0.03 mg L^{-1} respectively (n=106). Of the groundwater samples containing methane 73 % had a concentration of <1 mg L^{-1}. Ethane was detected in 23 samples with mean and median concentrations of 35 ± 71 µg L^{-1} and 2 µg L^{-1} respectively. Methane concentrations were significantly higher in old waters compared to other waters ($p<0.05$) and old waters were often associated with high cell counts (Figure 4) and low concentrations of oxygen (Fig. 9a), sulfate (Fig. 3b) and nitrate (Supplementary Data 1). Isotopic analysis supported widespread microbial methane production with $\delta^{13}C_{CH4}$ < -60 ‰, $\delta^{2}H_{CH4}$ < -200 ‰ (Supplementary Fig. 1a). Especially in older waters the $\delta^{13}C_{CO2}$ > +10 ‰ strongly supported methane production via CO_2 reduction (Fig. 3c, Supplementary Fig. 1b), which is corroborated by the presence of high relative sequence abundances of hydrogenotrophic methanogens, while aceticlastic and methylotrophic methanogens were less abundant in older waters and largely occurred in younger and intermediate waters, as well as numerous sulfate-rich aquifers (see Supplementary Information sections below). A total of 17 samples with an average low methane concentration of 0.03 mg L^{-1} had increased $\delta^{13}C_{CH4}$ values of over –50 ‰ up to –22 ‰ (Fig. 3a) suggesting that methane oxidation has occurred. Methane oxidation apparently occurred in many groundwater samples that also showed sulfate reduction (Fig. 3a, b). In groundwaters with low concentrations of electron acceptors, the *in-situ* production of methane by methanogenesis becomes a thermodynamically favorable process. Methanogenesis is believed to proceed through two different major pathways, which are the reduction of inorganic carbon in the presence of hydrogen (hydrogenotrophic methanogenesis; Eq. (1)):

$$4\,H_2 + HCO_3^- + H^+ = CH_4 + 3\,H_2O \qquad (1)$$

or the fermentation of acetate (acetoclastic methanogenesis; Eq. 2):

$$CH_3COOH + H_2O = CH_4 + HCO_3^- + H^+ \qquad (2)$$

Sulfate

Sulfate was widespread in Alberta groundwater samples and often present in relatively high concentrations as compared to the average standard sulfate concentration (250 mg L^{-1} WHO). Natural sources of sulfate include sulfur mineral dissolution, atmospheric deposition, and sulfide oxidation from minerals[17]. Reduced sulfur in minerals such as pyrite or in organo-sulfur compounds are common in shales, coal seams and many other rock types. One hypothesis is that the oxidation of reduced sulfur (pyrite and/or organo-sulfur compounds), incorporated from the Albertan bedrock into the glacial deposit during glaciation, occurs in the till[18]. Oxygen dissolved in the recharging groundwater and gaseous oxygen created oxic conditions needed to convert reduced sulfur into sulfate. S- O- isotope ratios of sulfate have been investigated to further evaluate the possible origin of sulfate in the groundwater. The $\delta^{34}S_{SO4}$ values in groundwater ranged from –17.5 to + 65.2 ‰ with a mean value of +1.6 ‰ (n=89). The $\delta^{18}O_{SO4}$ values in groundwater ranged from –16.1 to + 15.3 ‰ with a mean value of – 1.4 ‰ (n=89). The highest concentrations of sulfate were found in sulfate-rich, old groundwater that had $\delta^{18}O_{SO4}$ values of below 0 ‰ and $\delta^{34}S_{SO4}$ values of below 0 ‰ (Fig. 3b, d), suggesting that indeed oxidation of sulfide minerals was the main sulfate source. It is known that the light isotopes ^{32}S and ^{16}O are preferentially metabolized during bacterial sulfate reduction, thus causing a decrease in sulfate concentrations while ^{34}S and ^{18}O become progressively enriched in the remaining sulfate. We show that many samples with low sulfate concentrations had increased $\delta^{34}S_{SO4}$ values > +10 ‰

suggesting that sulfate reduction has occurred in the aquifers (Fig. 3b). These waters also showed elevated $\delta^{18}O_{SO4}$ values and often high methane concentrations.

Oxygen

Highest dissolved oxygen (DO) concentrations were found in the freshly recharged (Ca^{2+}-Mg^{2+}-HCO_3^-) groundwater with an average concentration of 1.83 ± 2.35 mg L^{-1}, up to 7 mg L^{-1} approaching the saturation of oxygen in water, indicating mixed oxic-hypoxic conditions (Fig. 9a). Intermediate DO concentrations of 1.18 ± 1.76 mg L^{-1} were measured in intermediate, and sulfate-rich old groundwater samples (0.88 ± 1.29 mg L^{-1}), and lowest average DO of 0.51 ± 0.87 mg L^{-1} was associated with sulfate-poor, old water (Na^+-HCO_3^--Cl^-). At certain sites we observed low $\delta^{18}O_{O2}$ values (as low as +21 ‰), while also finding elevated O_2/Ar ratios (Fig. 9b). The lower $\delta^{18}O_{O2}$ and higher O_2/Ar ratios interestingly fell along a trend, consistent with the simulated addition of DO with a $\delta^{18}O$ value much lower than that of air-equilibrated water (dashed trend line in Fig. 9b). As an idealized example, if 0.5 mg L^{-1} of O_2 with $\delta^{18}O_{O2}$ equal to -20 ‰ were added to air-equilibrated water, the net effect would be to lower the $\delta^{18}O_{O2}$ by ~2 ‰ while increasing the O_2/Ar ratio by ~5 %, consistent with the slope of the dashed line in Fig. 9b.

Nitrogen compounds, iron, and manganese

In our study nitrate was only detected in younger groundwater, although it is not uncommon even in aquifers bearing older groundwater, pre 1953[19]. Nitrite was detected in 4 samples (<0.076 mg L^{-1}). The dissolved Fe and Mn concentrations in the groundwater samples are generally low, both mostly below 0.2 mg L^{-1} with maximum concentrations of 1.5 mg L^{-1} and 6.7 mg $L^{-1,}$ respectively.

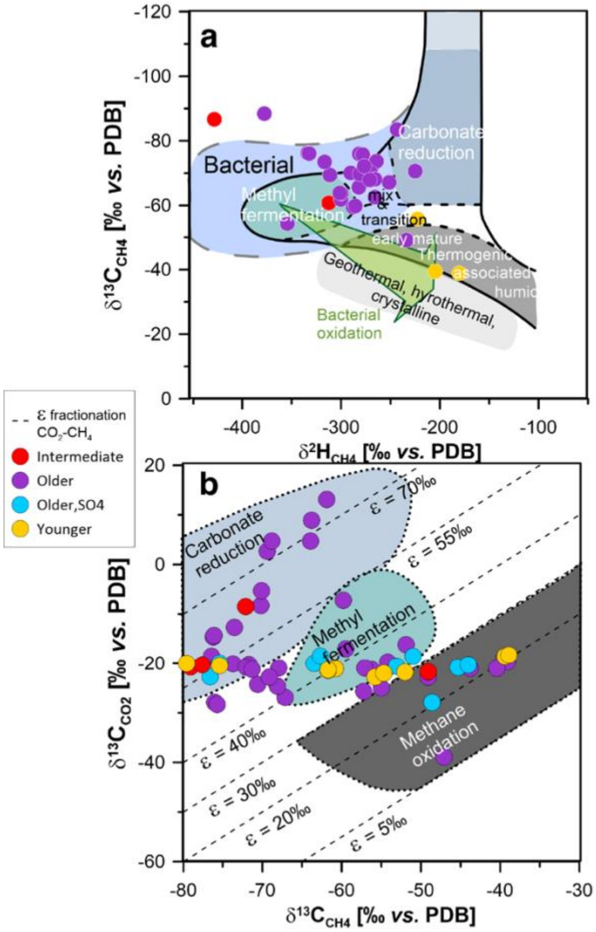

Supplementary Figure 1. Methane carbon isotope geochemistry. Classification of microbial and thermogenic gas by combination of $\delta^{13}C_{CH4}$ and $\delta^{2}H_{CH4}$ (a) and $\delta^{13}C_{CO2}$ and $\delta^{13}C_{CH4}$ (b).

Further results on section: Old groundwater contains biomass-rich microbial communities

Cell abundance

In addition to enumerating the cells in 78 samples (Fig. 4, Supplementary Data 2) we have estimated the number of cells that can be sustained by the missing dissolved oxygen concentration in the water samples (Supplementary Data 8). To estimate how much oxygen was maximally consumed, i.e., was missing, we subtracted the measured O_2 concentration from the O_2 saturation concentration. We estimated how many cells could have been sustained on this missing oxygen using previously published values for biomass per mol oxygen yields[20] and biomass per microbial cell[21]. The formula is included in the Supplementary Data 8. We found that the estimated cell number was always much higher than the counted cell number and thus the observed cell abundance could have been sustained by the missing oxygen concentration in all aquifers. In fact, even the measured O_2 concentration could have sustained the counted cell abundances in most aquifers indicating that oxygen was not limiting in these systems.

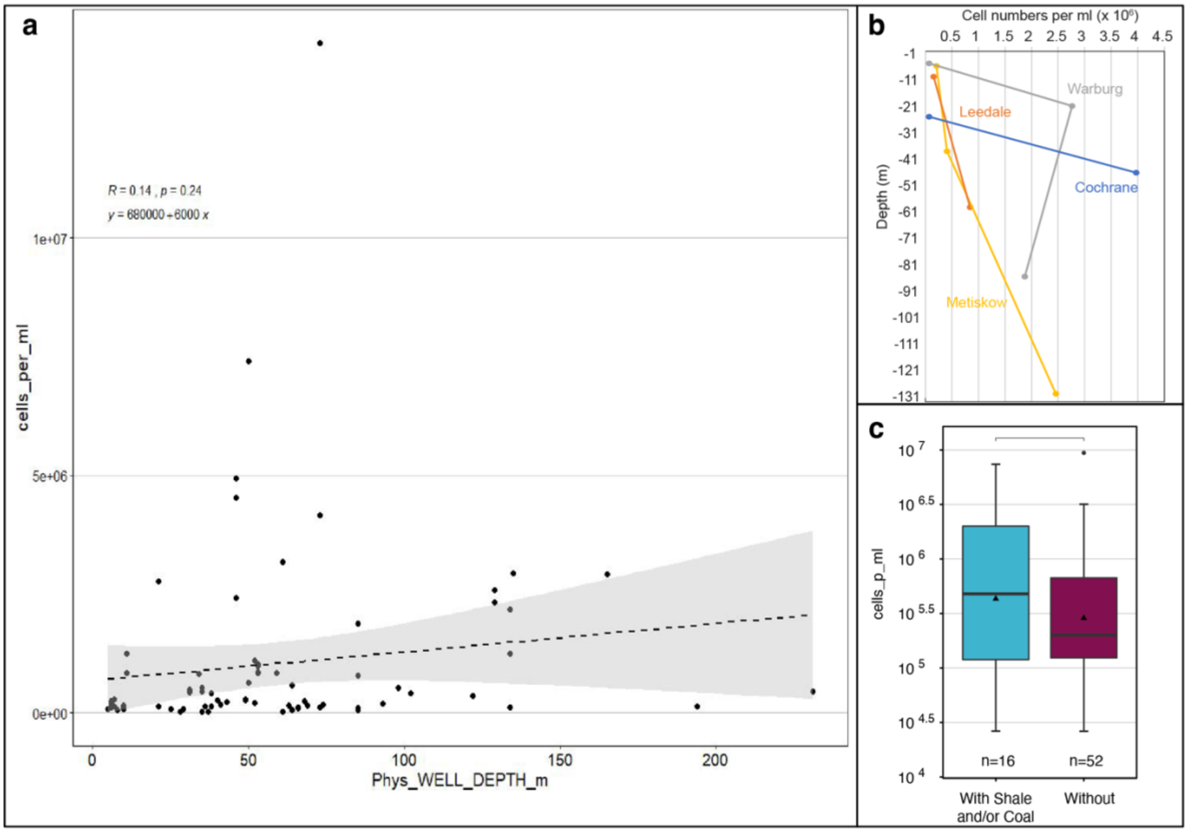

Supplementary Figure 2. Microbial cell numbers. **a** Cell abundance versus well depth across the entire dataset. Each dot represents one sample. The linear regression (dashed line, using Pearson's correlation, gray area shows standard error at 95% confidence) is not significant (R=0.14, p=0.24), but it was included to show that there is not a trend of decreasing cell numbers with increasing depth, if at all the data suggest an opposite trend. **b** Cell numbers versus well depth in selected aquifers for which multiple depth horizons were available to sample. **c** Cell abundance in wells that were completed into formations with or without coal or shale or both. Here too, the differences are not statistically significant (Wilcoxon rank sum test), but the data indicate that cell abundance may have been higher in energy-rich strata. Boxplots visualize data using upper and lower quartiles and whiskers (each representing 25% of the data), median (line), average (triangle) and outliers (dots).

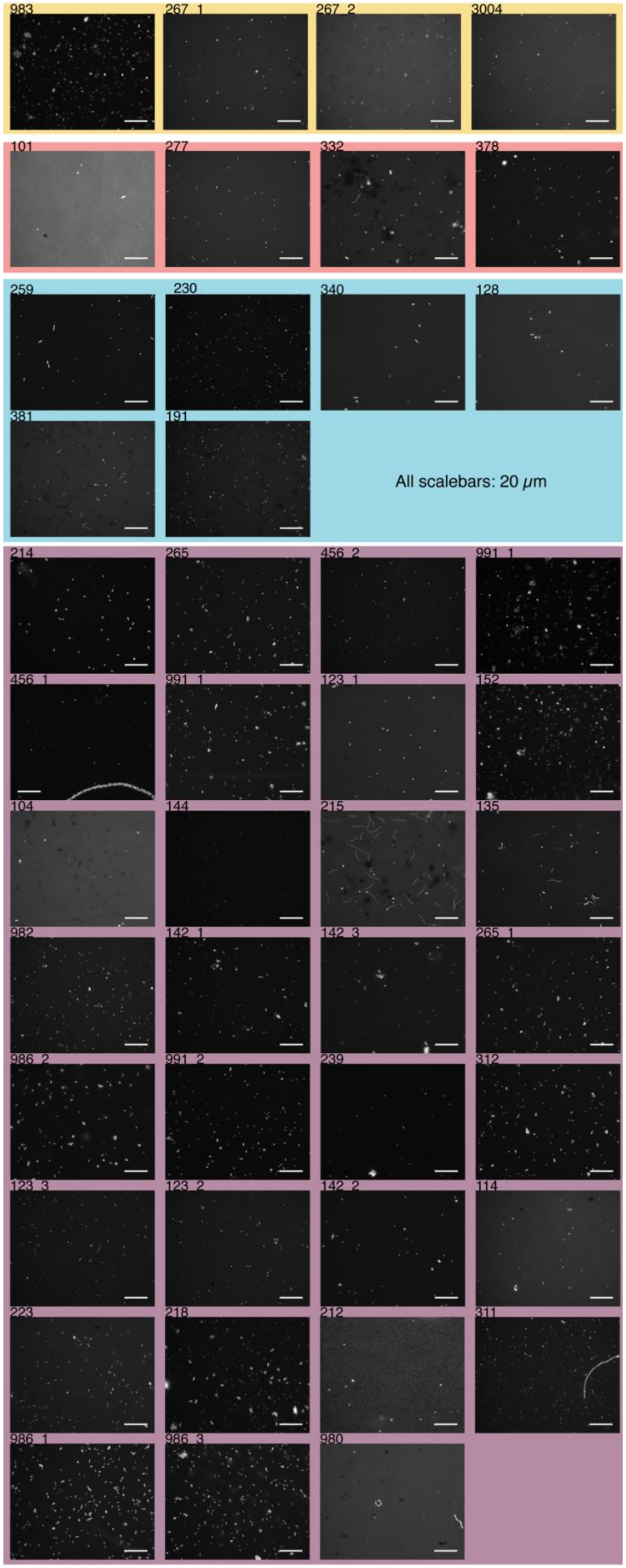

Supplementary Figure 3. Fluorescence micrographs. Cell numbers and morphologies present in groundwater samples, as detected with the nucleic acid stain DAPI. The background color refers to the four water ages (yellow: younger, red: intermediate, blue: older with sulfate, purple: older). Average cell size is similar in the different water types, indicating that increased cell numbers equal increased biomass. A high-resolution .tif image is available in the Raw Data folder. Each picture is representing one sample. Detailed cell counts obtained from multiple fields of, as well as the spread of values, reproducibility, and biological replicates is shown in Figure 4.

Microbial diversity and community structure

Like the alpha diversity metrics shown in Fig 4a, b, the evenness and estimated richness of the archaeal and bacterial communities decreased with increasing groundwater age (Supplementary Fig. 4). Any two samples shared on average 5.7 % of archaeal and 5.5 % of bacterial ASVs (Fig. 5c, d). There were, however, very different samples that shared no ASV and very similar samples that shared 93 % archaeal or 71.4 % bacterial or ASVs. The archaeal and bacterial community structure of all four water types is different yet overlapping (ANOSIM: R_{Arc}=0.29, p_{Arc}=0.001, R_{Bac}=0.33, p_{Bac}=0.001), The NMDS ordinations show a trend from young, via intermediate, to older waters, indicating substantial community turnover with age (Fig. 5c, d). However, some microorganisms are exchanged between aquifers, because around 1 % of all archaeal (32 ASV) and 3 % of all bacterial ASV (388 ASV) occurred in all water types. The patterns of ASV occurrence also indicate that intermediate and old waters are in exchange with young waters (Supplementary Fig. 5).

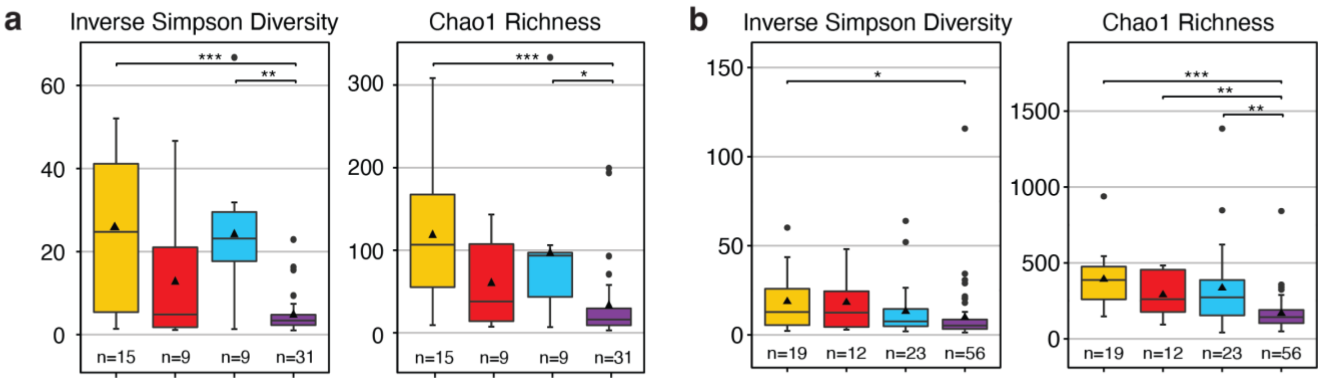

Supplementary Figure 4. Microbial diversity with water age. Archaeal **a** and bacterial **b** community evenness and estimated richness, given by Inverse Simpson Diversity and Chao1 richness, respectively. The decreasing evenness and richness indicate an increase in relative abundance of certain clades. This could mean the selective growth or the selective survival of certain key lineages. In our case, the selective growth or enrichment of key lineages is supported by an increase in overall microbial abundance (Fig. 4), constituting a bloom situation. Boxplots summarize data using upper and lower quartiles and whiskers (each representing 25% of the data), median (line), average (triangle) and outliers (dots). Significance was tested using a Wilcoxon rank sum test. Significance levels are: *: p<0.05; **: p<0.01; ***: p<0.001; uncorrected.

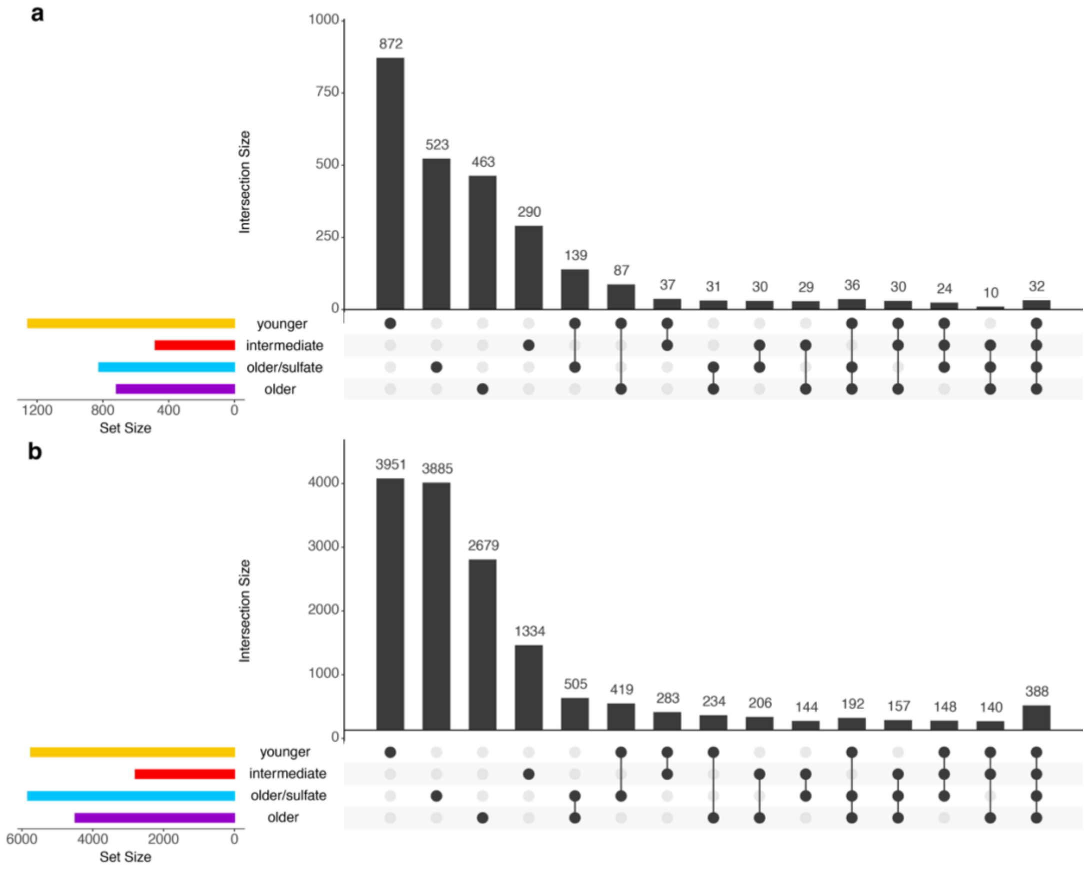

Supplementary Figure 5. Number of unique and shared amplicon sequence variants. Upset plot[22] showing **a** archaeal and **b** bacterial richness (number of amplicon sequence variants – ASV) that exclusively occur in the investigated water types and combination. The plot is to be read like a Venn diagram. The richness patterns are similar across domains with younger waters showing the highest richness, followed by older and intermediate waters. A core community of around 1 % of all archaeal (32 ASV) and 3 % of all bacterial ASV (388 ASV) occurred in all water types, suggesting the persistence of certain microbial populations in aquifers of different depths and ages.

Community variation

We investigated a selected set of environmental parameters based on their completeness, i.e., the maximum number of samples for which a parameter was available, and their potential to explain the variance observed in the ASV-based microbial community structure. We selected O_2, CO_2, methane, d^{13}C-DIC, calcium, hardness, magnesium, fluoride, pH, sodium, sulfate, and well depth. We used these parameters for an in-depth Redundancy Analysis (RDA, Fig. 5e, f; raw data folder) and statistical significance testing using ANOVA permutation tests[23]. The full model was highly significant for both domains and explained 25 % of archaeal and 18 % of bacterial variation (raw data folder). Upon investigating each parameter in a reduced model (partial RDA), we found that variation in archaeal community structure was explained by well depth and ^{13}C-DIC at low significance (Fig. 5e). Bacterial community structure was best explained by methane concentration, but well depth, hardness, calcium, and magnesium concentrations also explained a substantial part of the community variation (Fig. 5f).

Further results on section: Hydrogen as basal energy source in productive aquifers

Community composition

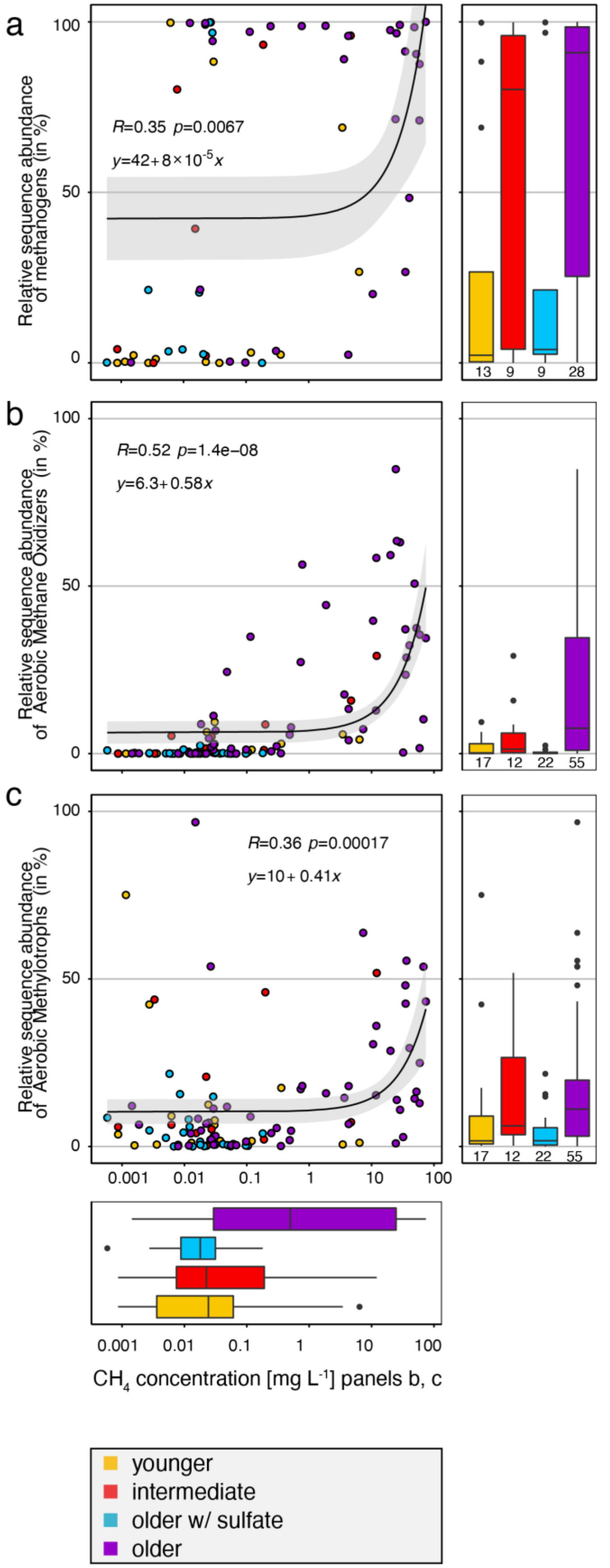

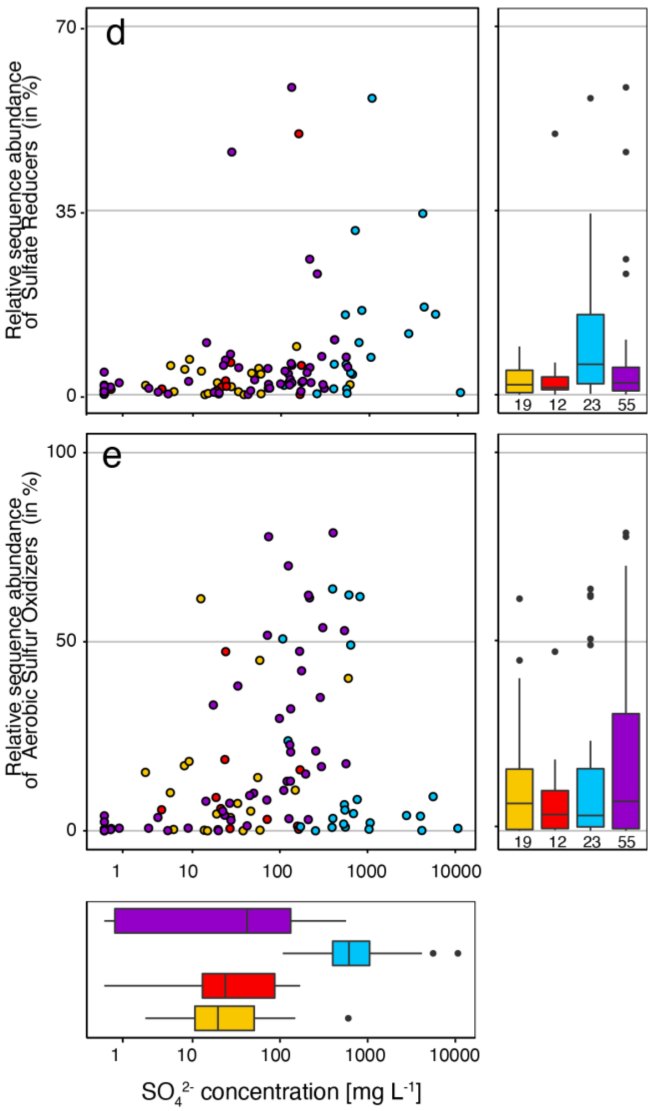

Supplementary Figure 6. Methane and sulfur cycling microbes. Relative abundance of **a** methanogens, **b** aerobic methanotrophs and **c** aerobic methylotrophs versus methane concentration (left panels). There is a significant positive correlation of methane-cycling clades with the concentration of methane (using Pearson's, linear regression with standard error bands at 95% confidence). **d** Sulfate reducers and **e** sulfur oxidizers versus sulfate are shown in the right panels. Sulfate reducers tend to be abundant in sulfate-rich waters, while sulfide oxidizers are most abundant in waters with intermediate sulfate concentrations. Concentrations on the x-axis are shown on pseudo-log_{10} scale to include the samples in which the compound was not detected (zero). Boxplots summarize data using upper and lower quartiles and whiskers (each representing 25% of the data), median (line) and outliers (dots).

Methanogens

The large majority of archaeal ASV affiliated with lineages known to perform methanogenesis (Fig. 6a, Supplementary Fig. 7). Hydrogenotrophic methanogens were found in all water types and in all samples with few exceptions (GW128, GW3002, GW220, GW104, and GW259). Here, the methanogenic community consisted of methylotrophic clades, mainly *Methanolobus sp*. and *Methanomassiliicoccales*. Overall, hydrogenotrophic *Methanobacterium sp*. and *Methanoregula sp.*, as well as aceticlastic *Methanosaeta sp*. were the most abundant methanogens in the datasets. Although methylotrophic clades occurred at high relative abundances (> 50 %) in four samples of mature waters, they were generally more abundant in younger and sulfate-rich waters. Methanogenic archaea apparently tolerated low concentrations of oxygen that were present in most aquifers (Fig. 9a).

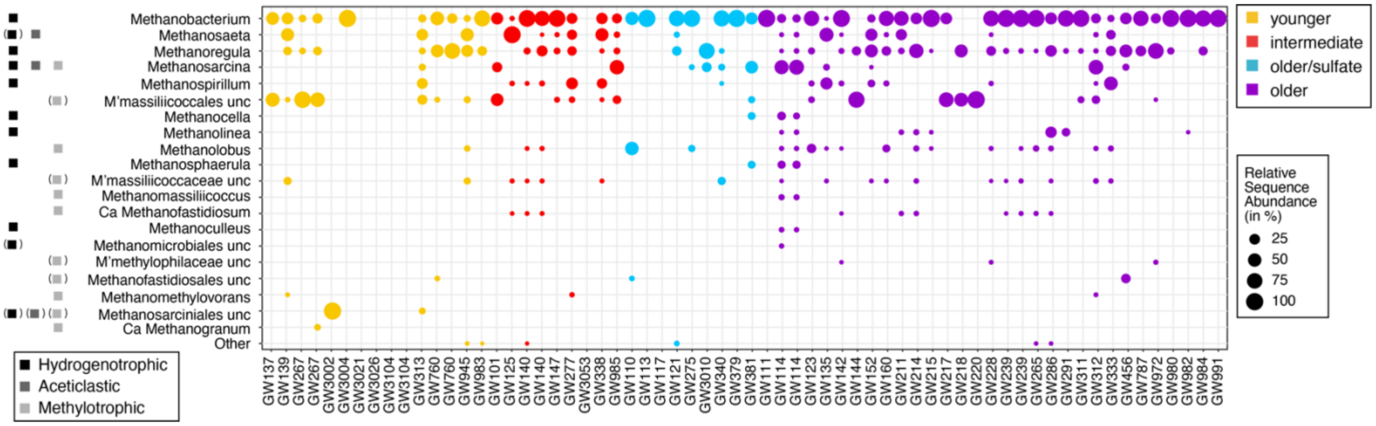

Supplementary Figure 7. Methanogenic microbes. Relative sequence abundance of genus-level methanogenic clades based on 16S rRNA amplicon sequence variants (ASV). Clades marked by unc represent genus level clades without cultured isolates, here the phylogenetic level with the closest cultured isolate is given. The different methanogenic capabilities hydrogenotrophic, aceticlastic and methylotrophic are shown as squares. Squares in brackets represent capabilities that are likely, yet not confirmed for the respective clade. Note: The shown relative abundance was calculated using only methanogenic clades and thus can be used to interpret the importance of each lineage within this guild rather than its importance in the whole community.

Methanotrophs, methylotrophs and short-chain alkane oxidizers

ASVs affiliating with *Methanoperedenaceae* – known to perform the anaerobic oxidation of methane (AOM) – almost exclusively occurred in old waters. In three aquifers (GW218, GW111 and GW456) these methanotrophs accounted for over 50 % of the archaeal community indicating that AOM may be of significance in these aquifers (Fig. 10, Supplementary Data 3). ASVs affiliating with clades that are known to perform aerobic metabolisms accounted for most of bacterial diversity (Supplementary Fig. 8). Aerobic methylotrophic *Methylophilaceae* occurred in all waters except those with sulfate, while *Methylomonadaceae* predominantly occurred in older waters. *Methylibium sp.* and obligate alkane-degrading *Alkanindiges sp* indicate that short-chain alkanes and aromatics could serve as a carbon and energy source in some of the aquifers[24,25]. Despite the large overall diversity in methane-cycling clades each investigated aquifer contained signatures of at least one methanogen, methanotroph and methylotroph, supporting the potential for methane remediation and indicating that these aquifers are biofilters removing methane before it outgasses to the atmosphere. The presence of aerobic and anaerobic

methanotrophs was reported for several deep bedrock sites[26]. Their coexistence and the resulting syntrophy as well as functional redundancy suggest that methane cycling is an essential ecosystem function in high productivity aquifers.

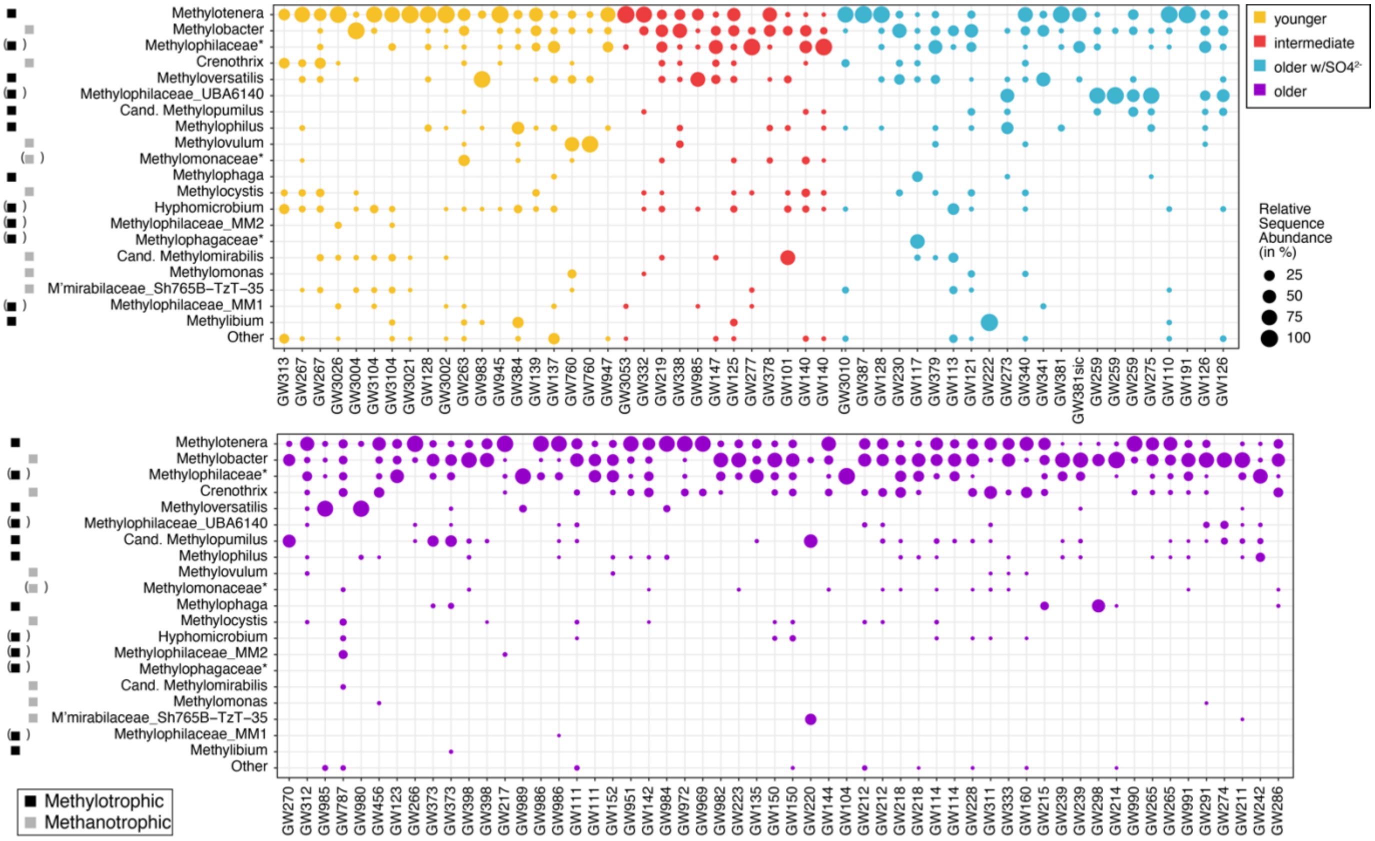

Supplementary Figure 8. Methano- and methylotrophic microbes. Relative sequence abundance of genus-level aerobic methano- and methylotrophic clades based on 16S rRNA amplicon sequence variants (ASV). Clades marked by an asterisk represent genus level clades without cultured isolates, here the phylogenetic level with the closest cultured isolate is given. The different methanotrophic capabilities, methanotrophic and methylotrophic, are shown as squares. Squares in brackets represent capabilities that are likely, yet not confirmed for the respective clade. Note: The shown relative abundance was calculated using only methano- and methylotrophic clades and thus can be used to interpret the importance of each lineage within these guilds rather than its importance in the whole community.

Sulfate reducers

Older sulfate-rich groundwaters had the highest relative sequence abundance of sulfate reducers (Supplementary Fig. 9). *Desulfomicrobium* was the second most abundant genus occurring mainly in old waters, unlike *Desulfocapsa*, a clade known for sulfur disproportionation, which occurred on moderate to high relative abundance in many samples (66 of 108) across all water types. Sequences affiliating with the genus *Desulfomonas* known to perform sulfur reduction were also very widespread (found in 72 of 108 samples), except in young recharge waters. Overall, the aquifers apparently had a high functional redundancy harboring multiple sulfate-reducing populations. There were, however, some exceptions that were dominated by just one or few clades. In GW3104, GW378, GW969, GW218, GW298, GW117 and

GW 155 we detected between two and four populations likely involved in sulfate reduction, while GW3002 harbored only one sulfate reducer affiliating with an unclassified genus of *Desulfobulbaceae*.

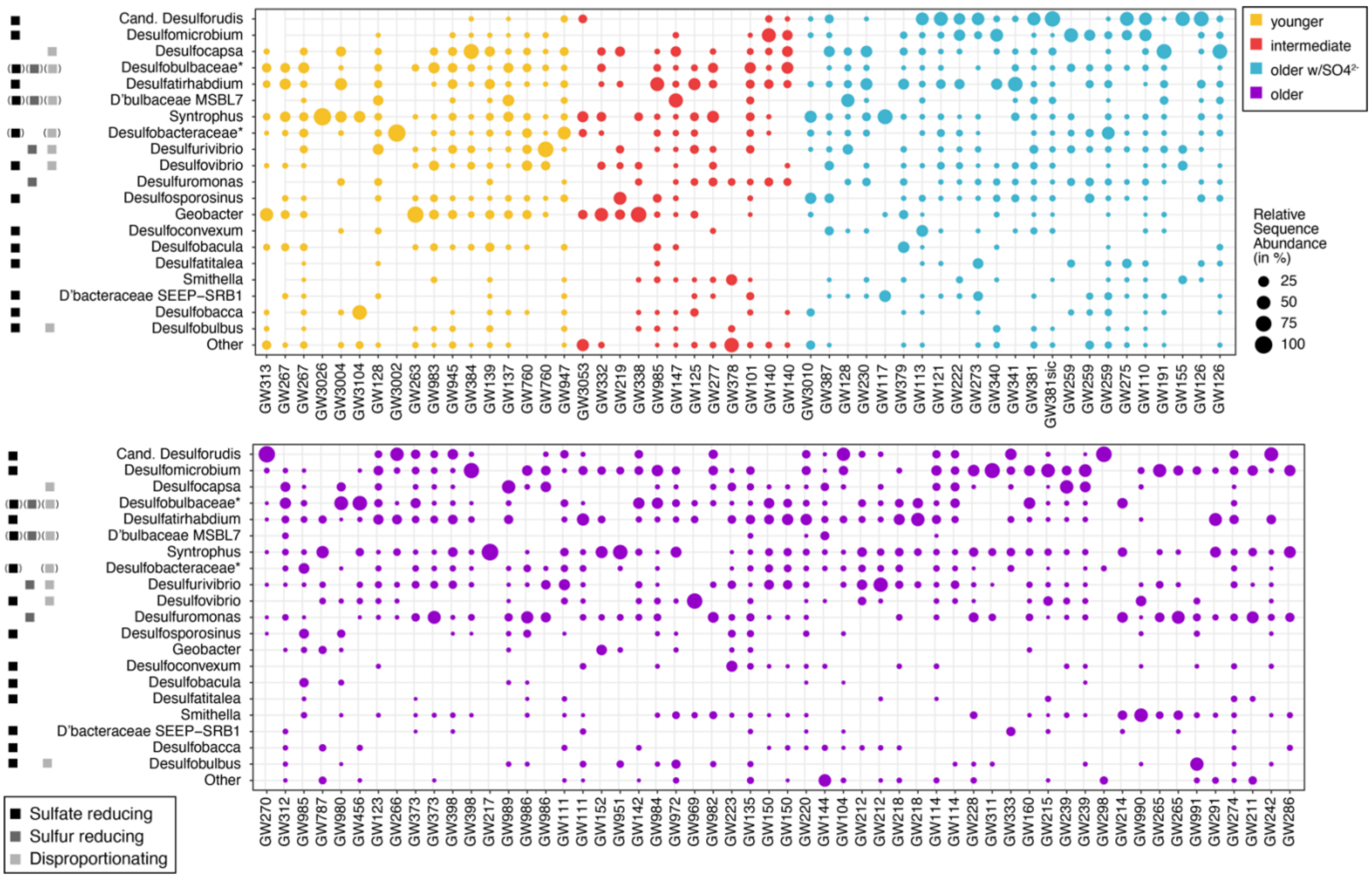

Supplementary Figure 9. Sulfate-reducing microbes. Relative sequence abundance of genus-level sulfate-reducing clades based on 16S rRNA amplicon sequence variants (ASV). Clades marked by an asterisk represent genus level clades without cultured isolates, here the phylogenetic level with the closest cultured isolate is given. The different sulfur cycling capabilities are shown as squares. Squares in brackets represent capabilities that are likely, yet not confirmed for the respective clade. Note: The shown relative abundance was calculated using only sulfate-reducing clades and thus can be used to interpret the importance of each lineage within this guild rather than its importance in the whole community. *Geobacter*, *Syntrophus* and *Smithella* were included because they are close relatives and/or known to live syntrophically with hydrogen scavengers.

Sulfur oxidizers

Putative sulfur oxidizers were very widespread in the studied aquifers, including facultatively anaerobic *Sulfuricurvum sp.* which are known to oxidize sulfur with either oxygen or nitrate, and are also able to use hydrogen as an electron donor[27]. Some *Thiobacillus sp.* can anaerobically oxidize pyrite using nitrite as electron acceptor[28], and may also use hydrogen, sulfur compounds or iron as electron donors[29]. *Thiomicrorhabdus* have been shown to be versatile sulfur oxidizers in coal mine shafts[30]. In two of the aquifers, they constituted 100 % of the sulfur oxidizers present (Supplementary Fig. 10, GW3026 and GW3004). *Sulfurimonas* is also known from subsurface ecosystems[31] and may oxidize sulfur compounds using nitrate, which was however found in few groundwater samples. *Sulfurimonadaceae* were mainly found in younger or older waters, while *Sulfuricellaceae* occurred in all groundwater types at low

abundance. Sulfate-rich waters had a sulfur-isotopic signature indicating oxidation-derived sulfate (Fig. 3b, d) of abiotic or biotic origin.

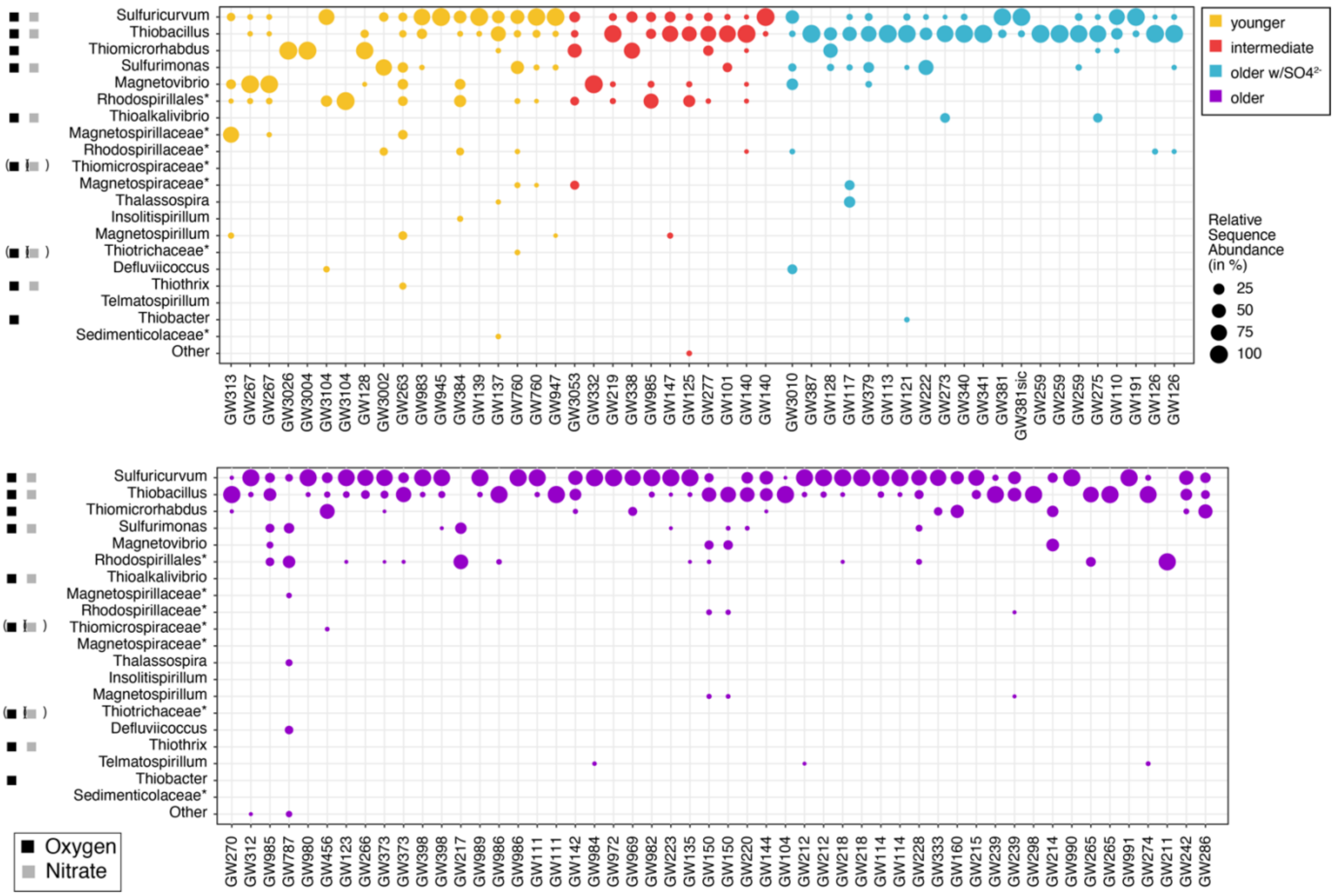

Supplementary Figure 10. Sulfur-oxidizing microbes Relative sequence abundance of genus-level sulfur-oxidizing clades based on 16S rRNA amplicon sequence variants (ASV). Clades marked by an asterisk represent genus level clades without cultured isolates, here the phylogenetic level with the closest cultured isolate is given. The different electron acceptor for sulfur oxidation are shown as squares. Squares in brackets represent capabilities that are likely, yet not confirmed for the respective clade. Note: The shown relative abundance was calculated using only thiotrophic clades and thus can be used to interpret the importance of each lineage within this guild rather than its importance in the whole community. *Rhodospirillales* and other clades that comprise non-sulfur-oxidizing organisms were included because they are close relatives.

Further results on section: <u>Aquifers harbor microbes affiliating with aerobic and facultatively anaerobic lineages</u>

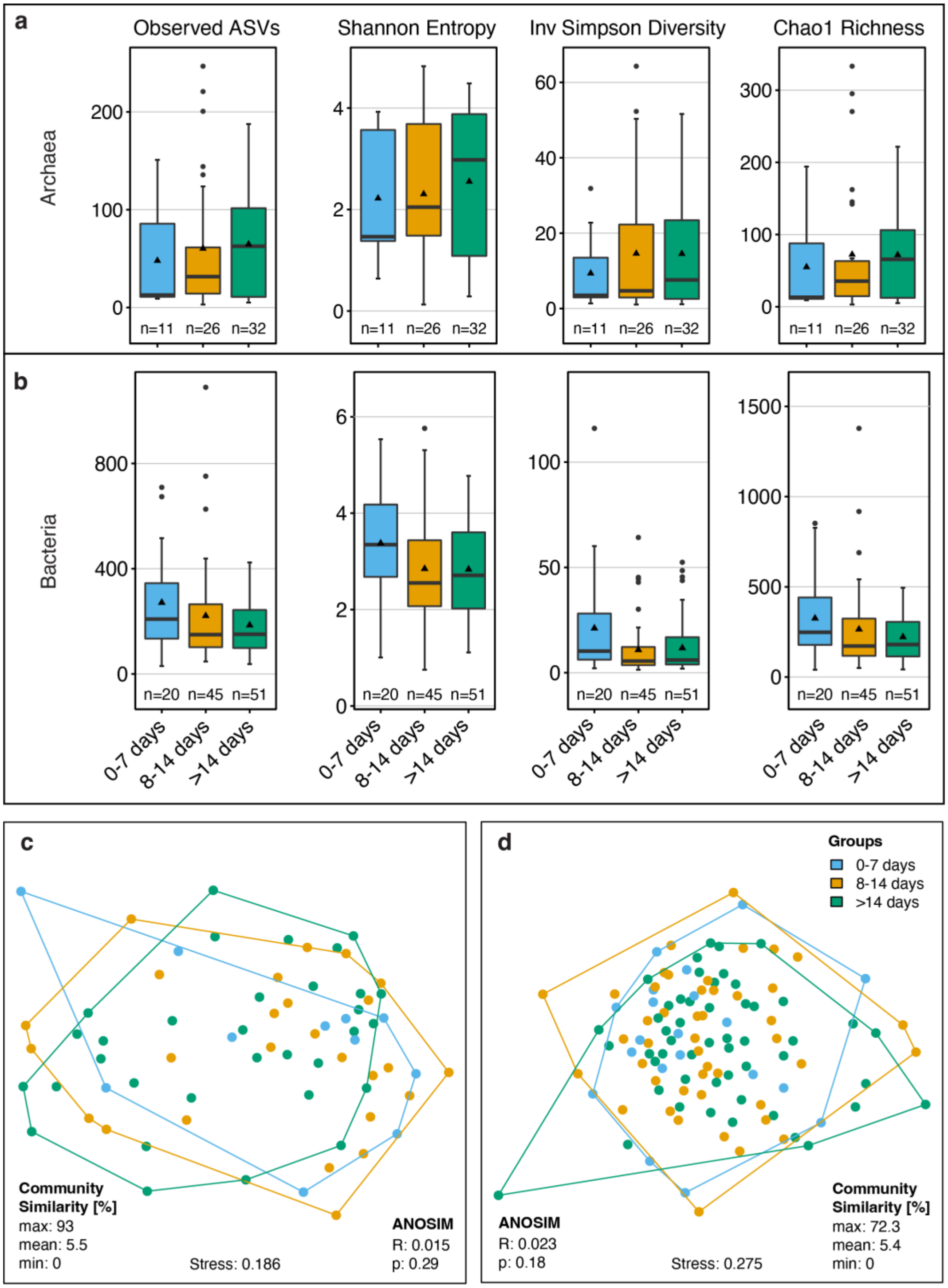

Supplementary Figure 11. Community structure vs sample storage time. Archaeal **a** and bacterial **b** alpha diversity grouped based on storage duration. Boxplots visualize data using upper and lower quartiles and whiskers (each representing 25% of the data), median (line), average (triangle) and outliers (dots). Archaeal **c** and bacterial **d** beta diversity using the same groups. Wilcoxon (**a**, **b**) and ANOSIM and Procrustes tests (**c**, **d**) were not significant, i.e. alpha and beta diversity as well as composition (not shown) were similar in all three groups.

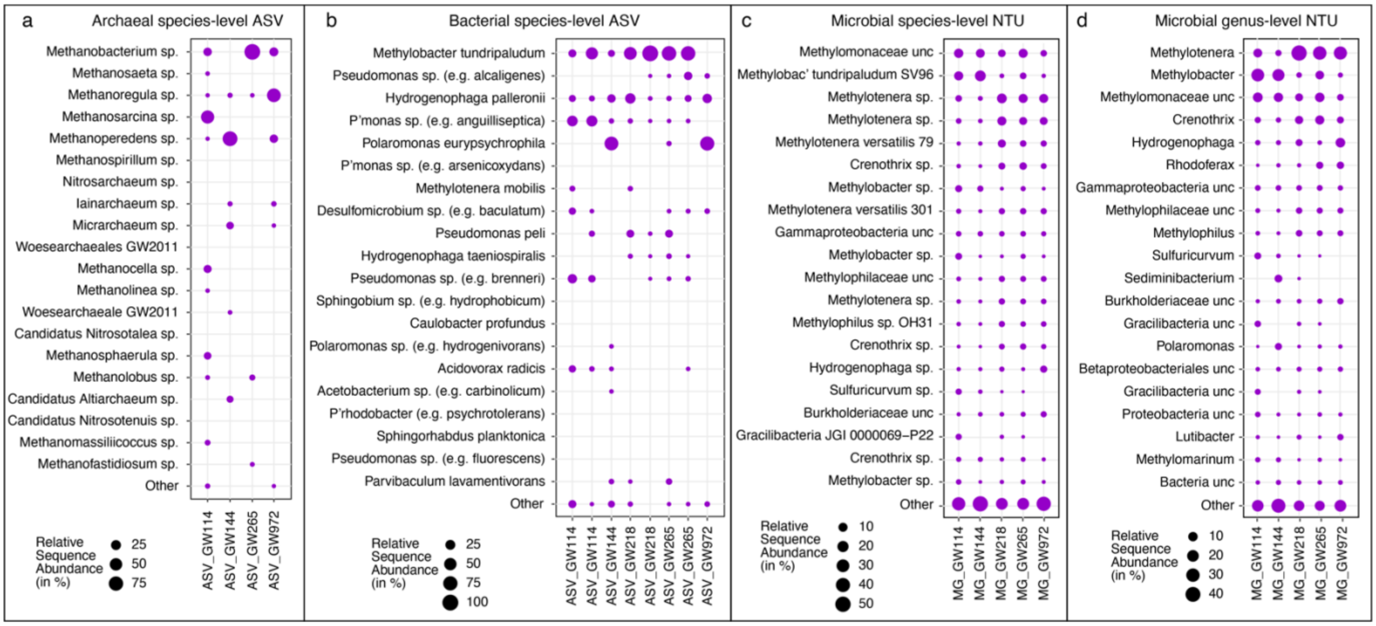

Supplementary Fig. 12. Metabarcoding- and metagenome-based microbial community composition. The species-level microbial community composition based on **a** archaeal and **b** bacterial 16S rRNA gene amplicon sequence variants (ASVs) is very similar to the **c** species- and **d** genus-level composition based on metagenome-derived nearest taxonomic units (NTUs). NTUs are metagenomic 16S rRNA gene sequence short reads mapped against a SILVA small subunit reference tree using the tool phyloFlash[32]. The top 20 most abundant lineages are shown. The community composition in **a**-**d** is very similar to the ASV-based genus-level composition shown in the main manuscript, as well as the composition of full-length 16S rRNA gene sequences reconstructed from the metagenomes (Supplementary Data 6). This shows that the presented trends in ASV-based community composition are reliable and reproducible on different phylogenetic levels as well as with different sequencing methods. For the **a** archaeal and **b** bacterial species-level analysis we reanalyzed the amplicon raw reads using the *species annotation* option of DADA2[33], and used only those ASV that were classified to species level. sp. represents unclassified species-level lineages in a known genus, unc represents unclassified species-level lineages in unclassified genera or higher phylogenetic levels. Some species-level clades were not found in these samples, but are listed nevertheless, because we used the 20 top clades of the overall dataset for consistency. Specific epithets are shown in brackets when ASV annotation was not reliably distinguishing between two or more closely related species. Samples that are listed twice are biological replicates from different years. Our results suggest that especially in the archaeal domain a lot of species-level novelty awaits discovery.

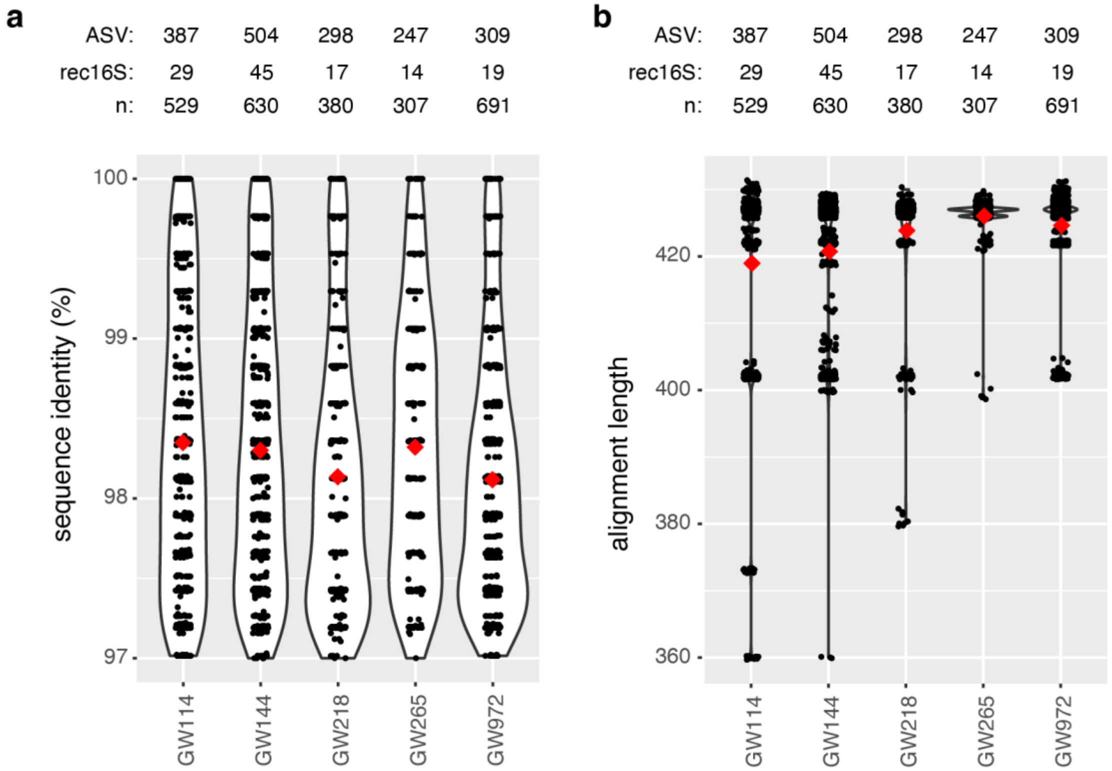

Supplementary Fig. 13. Sequence similarity between amplicon sequence variants (ASV) and full length 16S rRNA gene sequences. We used blastn to map ASVs to full length 16S rRNA gene sequences reconstructed from the metagenomes of corresponding samples. Many ASVs, including those of abundant key lineages, showed very high sequence identity **a** with 16S sequences reconstructed from the metagenomes. In most cases the alignment length **b** was equal to the length of the ASV sequences. This shows that many ASVs were highly similar and sometimes identical (but shorter) fragments of the metagenome-derived 16S rRNA gene sequences. This corroborates the reliability of both methods. **a** Each black dot is a blastn comparison, red diamonds are average values. The values above the graphs show the number of unique ASVs, the number of unique reconstructed 16S rRNA genes (rec16S), and the number of matches (n) between them. The number of matches is considerably higher than ASV or rec16S numbers, because several closely related ASVs (which often differ in just few bases) can match to the same rec16S. The full output is provided in Supplementary Data 7.

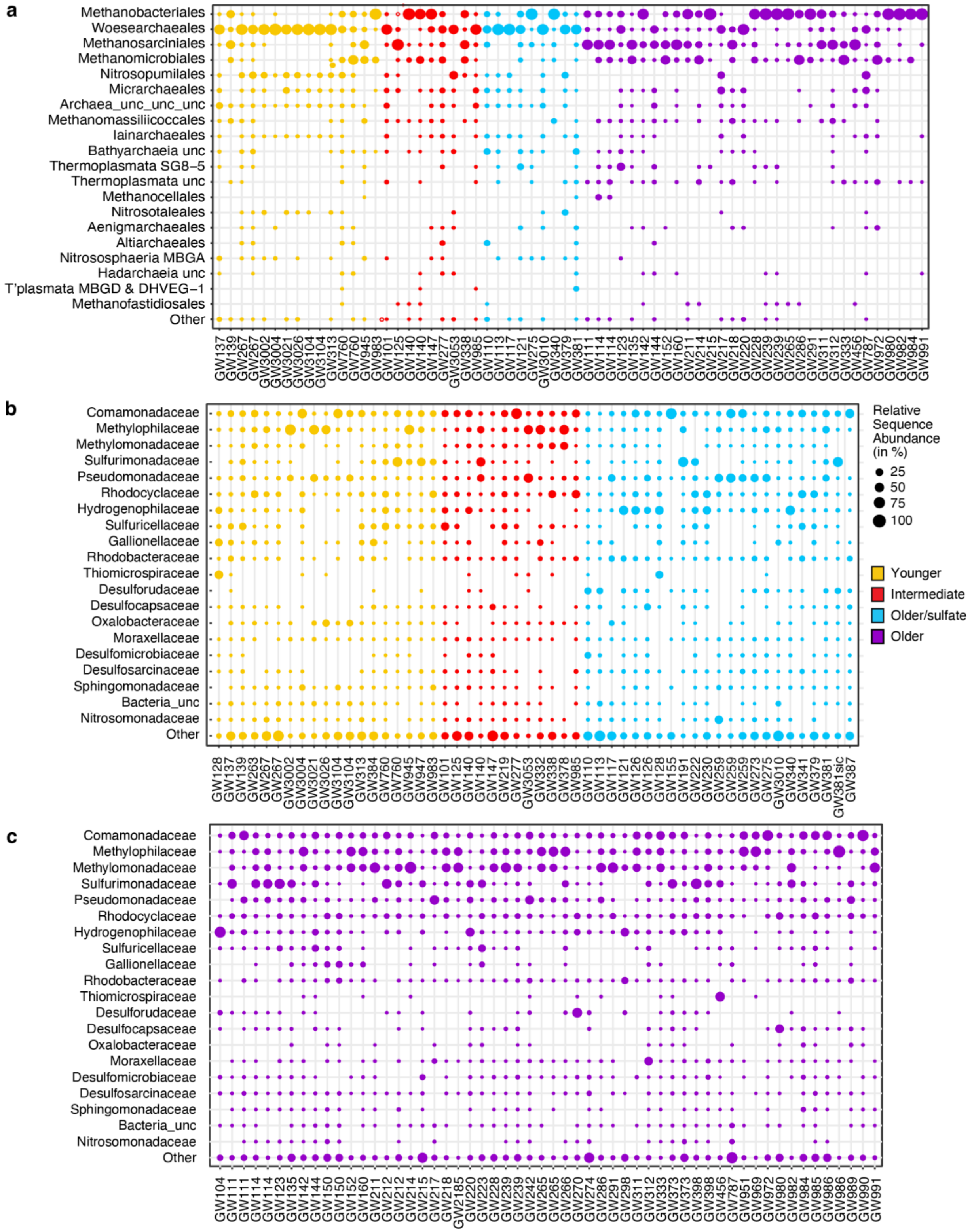

Supplementary Figure 14. Most abundant microbial clades. Relative sequence abundance of the 20 most abundant **a** archaeal order-level and **b, c** bacterial family-level clades based on 16S rRNA gene variable regions v6-v9 (*Archaea*) or v3-v4 (*Bacteria*).

Further results on section: Microbial production of dark oxygen via dismutation

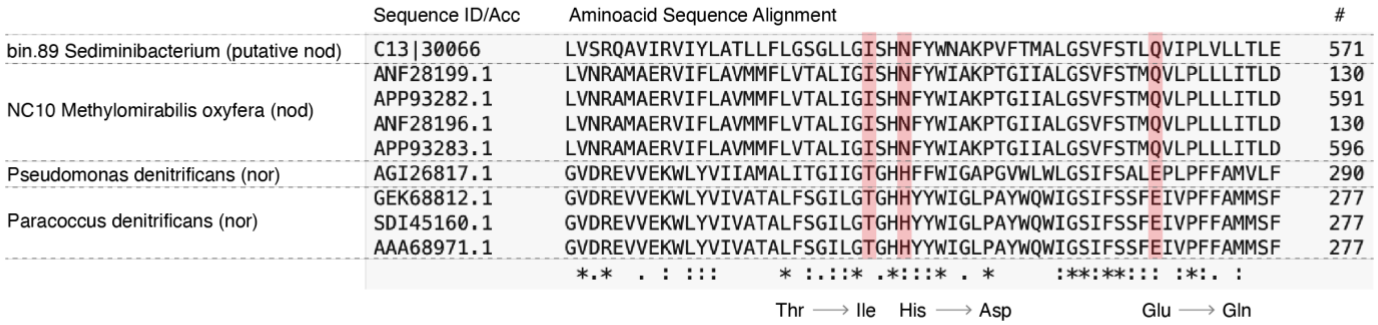

Supplementary Figure 15. Alignment of *nod* and *nor* gene sequences. The top row shows the amino acid sequence of the nitric oxide dismutase (*nod*) gene we found in the *Sediminibacterium* MAG of sample GW144. It shows the same diagnostic substitutions than the *nod* gene of the known NO-dismutating microbe *Methylomirabilis oxyfera*, which distinguish the gene from the related nitric oxide reductase (*nor*) shown in the lower half. The full alignment is provided in the raw data folder.

Supplementary References

1. Alberta Energy Regulator. *Alberta energy outlook - ST98 executive summary*. (2020).
2. Lyster, S. & Andriashek, L. *Geostatistical Rendering of the Architecture of Hydrostratigraphic Units within the Paskapoo Formation, Central Alberta*. *ERCB/AGS Bulletin* **August**, (Energy Resources Conservation Board/Alberta Geological Survey, 2012).
3. Dawson, F. M., Kalkreuth, W. D. & Sweet, A. R. *Stratigraphy and coal resource potential of the Upper Cretaceous to Tertiary strata of northwestern Alberta*. *Bulletin - Geological Survey of Canada* **466**, (1994).
4. Hamblin, A. P. *The Horseshoe Canyon Formation in Southern Alberta: Surface and subsurface stratigraphic architecture, sedimentology, and resource potential*. *Bulletin - Geological Survey of Canada* (2004). doi:10.4095/215068
5. Grasby, S. E., Chen, Z., Hamblin, A. P., Wozniak, P. R. J. & Sweet, A. R. Regional characterization of the Paskapoo bedrock aquifer system, southern Alberta. *Can. J. Earth Sci.* **45**, 1501–1516 (2008).
6. Meyboom, P. Geology and groundwater resources of the Milk River sandstone in southern Alberta. in 1–87 (Research Council of Alberta, 1960).
7. Rosenthal, L. R. P. Upper Cretaceous Wapiabi and Belly River formations. (McMaster University, 1984).
8. Pétré, M.-A., Rivera, A., Lefebvre, R., Hendry, M. J. & Folnagy, A. J. B. A unified hydrogeological conceptual model of the Milk River transboundary aquifer, traversing Alberta (Canada) and Montana (USA). *Hydrogeol. J.* **24**, 1847–1871 (2016).
9. Prior, G. J. *et al.* Bedrock geology of Alberta. *Alberta Energy Regulator, Alberta Geological Survey* scale 1:1,000,000 (2013).
10. Dalton, M. G. & Upchurch, S. B. Interpretation of hydrochemical facies by factor analysis. *Ground Water* **16**, 228–233 (1978).
11. Piper, A. M. A graphic procedure in the geochemical interpretation of water-analyses. *Trans. Am. Geophys. Union* **25**, 914–928 (1944).
12. Vogel, J. C. Carbon-14 dating of groundwater. in *Isotope Hydrology 1970* 225–239 (IAEA, 1970).
13. Reimer, P. J., Brown, T. A. & Reimer, R. W. Discussion: Reporting and calibration of post-bomb 14C data. *Radiocarbon* **46**, 1299–1304 (2004).
14. Stuiver, M. & Polach, H. A. Discussion: Reporting of 14C data. *Radiocarbon* **19**, 355–363 (1977).
15. Crann, C. A. *et al.* First status report on radiocarbon sample preparation techniques at the A.E.

Lalonde AMS Laboratory (Ottawa, Canada). *Radiocarbon* **59**, 695–704 (2017).
16. Mook, W. G. & van der Plicht, J. Reporting 14C activities and concentrations. *Radiocarbon* **41**, 227–239 (1999).
17. Krouse, H. R. & Mayer, B. Sulphur and oxygen isotopes in sulphate BT - environmental tracers in subsurface hydrology. in (eds. Cook, P. G. & Herczeg, A. L.) 195–231 (Springer US, 2000). doi:10.1007/978-1-4615-4557-6_7
18. Hendry, M. J., Cherry, J. A. & Wallick, E. I. Origin and distribution of sulfate in a fractured till in southern Alberta, Canada. *Water Resour. Res.* **22**, 45–61 (1986).
19. Rodvang, S. J., Mikalson, D. M. & Ryan, M. C. Changes in ground water quality in an irrigated area of southern Alberta. *J. Environ. Qual.* **33**, 476–487 (2004).
20. Garcia-Ochoa, F., Gomez, E., Santos, V. E. & Merchuk, J. C. Oxygen uptake rate in microbial processes: An overview. *Biochem. Eng. J.* **49**, 289–307 (2010).
21. Fagerbakke, K., Heldal, M. & Norland, S. Content of carbon, nitrogen, oxygen, sulfur and phosphorus in native aquatic and cultured bacteria. *Aquat. Microb. Ecol.* **10**, 15–27 (1996).
22. Conway, J. R., Lex, A. & Gehlenborg, N. UpSetR: an R package for the visualization of intersecting sets and their properties. *Bioinformatics* **33**, 2938–2940 (2017).
23. Borcard, D., Legendre, P. & Drapeau, P. Partialling out the spatial component of ecological variation. *Ecology* **73**, 1045–1055 (1992).
24. Nakatsu, C. H. *et al.* Methylibium petroleiphilum gen. nov., sp. nov., a novel methyl tert-butyl ether-degrading methylotroph of the Betaproteobacteria. *Int. J. Syst. Evol. Microbiol.* **56**, 983–989 (2006).
25. Klein, A. N., Frigon, D. & Raskin, L. Populations related to Alkanindiges, a novel genus containing obligate alkane degraders, are implicated in biological foaming in activated sludge systems. *Environ. Microbiol.* **9**, 1898–1912 (2007).
26. Kietäväinen, R. & Purkamo, L. The origin, source, and cycling of methane in deep crystalline rock biosphere. *Front. Microbiol.* **6**, 725 (2015).
27. Kodama, Y. & Watanabe, K. Sulfuricurvum kujiense gen. nov., sp. nov., a facultatively anaerobic, chemolithoautotrophic, sulfur-oxidizing bacterium isolated from an underground crude-oil storage cavity. *Int. J. Syst. Evol. Microbiol.* **54**, 2297–2300 (2004).
28. Juncher Jørgensen, C., Jacobsen, O. S., Elberling, B. & Aamand, J. Microbial oxidation of pyrite coupled to nitrate reduction in anoxic groundwater sediment. *Environ. Sci. Technol.* **43**, 4851–4857 (2009).
29. Drobner, E., Huber, H. & Stetter, K. Thiobacillus ferrooxidans, a facultative hydrogen oxidizer. *Appl. Environ. Microbiol.* **56**, 2922–2923 (1990).
30. Kadnikov, V. V *et al.* Sulfur-oxidizing bacteria dominate in the water from a flooded coal mine shaft in Kuzbass. *Microbiology* **88**, 120–123 (2019).
31. Lahme, S. *et al.* Comparison of sulfide-oxidizing Sulfurimonas strains reveals a new mode of thiosulfate formation in subsurface environments. *Environ. Microbiol.* **22**, 1784–1800 (2020).
32. Gruber-Vodicka, H. R., Seah, B. K. B. & Pruesse, E. phyloFlash: Rapid Small-Subunit rRNA Profiling and Targeted Assembly from Metagenomes. *mSystems* **5**, e00920-20 (2020).
33. Callahan, B. J. *et al.* DADA2: High-resolution sample inference from Illumina amplicon data. *Nat. Methods* **13**, 581–583 (2016).
