# Supplementary material for: Hydrogen and dark oxygen drive microbial productivity in diverse groundwater ecosystems
Source: Nat Commun. 2023 Jun 13;14:3194. doi: 10.1038/s41467-023-38523-4 (PMC10264387; doi:10.1038/s41467-023-38523-4)
Supplement: Supplementary file 3 — Description of Additional Supplementary Files [file 41467_2023_38523_MOESM3_ESM.pdf]

## Description of Additional Supplementary Files:

**Supplementary Data 1.** Physical, chemical, isotopic, and microbiological data of the studied GOWN wells

**Supplementary Data 2.** Fluorescence microscopy-based cell counts

**Supplementary Data 3.** Sequence abundance of archaeal 16S rRNA gene amplicon sequence variants

**Supplementary Data 4.** Sequence abundance of bacterial 16S rRNA gene amplicon sequence variants

**Supplementary Data 5.** Gene annotations and properties of metagenome-assembled genomes

**Supplementary Data 6.** Metagenome-derived reconstructed full-length 16S rRNA gene sequences

**Supplementary Data 7.** Sequence similarity between ASV and reconstructed full-length 16S rRNA genes

**Supplementary Data 8.** Estimated microbial oxygen consumption

**Supplementary Data 9.** Amino acid sequences of dismutase genes

**Supplementary Data 10.** Archaeal ASV based diversity indices

**Supplementary Data 11.** Bacterial ASV based diversity indices

**Supplementary Data 12.** Nucleotide sequences and taxonomy of archaeal ASVs

**Supplementary Data 13.** Nucleotide sequences and taxonomy of bacterial ASVs

**Supplementary Data 14.** Figure raw data and R scripts for amplicon sequence analyses
