# Supplementary figures and images for: Hydrogen and dark oxygen drive microbial productivity in diverse groundwater ecosystems
Source: Nat Commun. 2023 Jun 13;14:3194. doi: 10.1038/s41467-023-38523-4 (PMC10264387; doi:10.1038/s41467-023-38523-4)

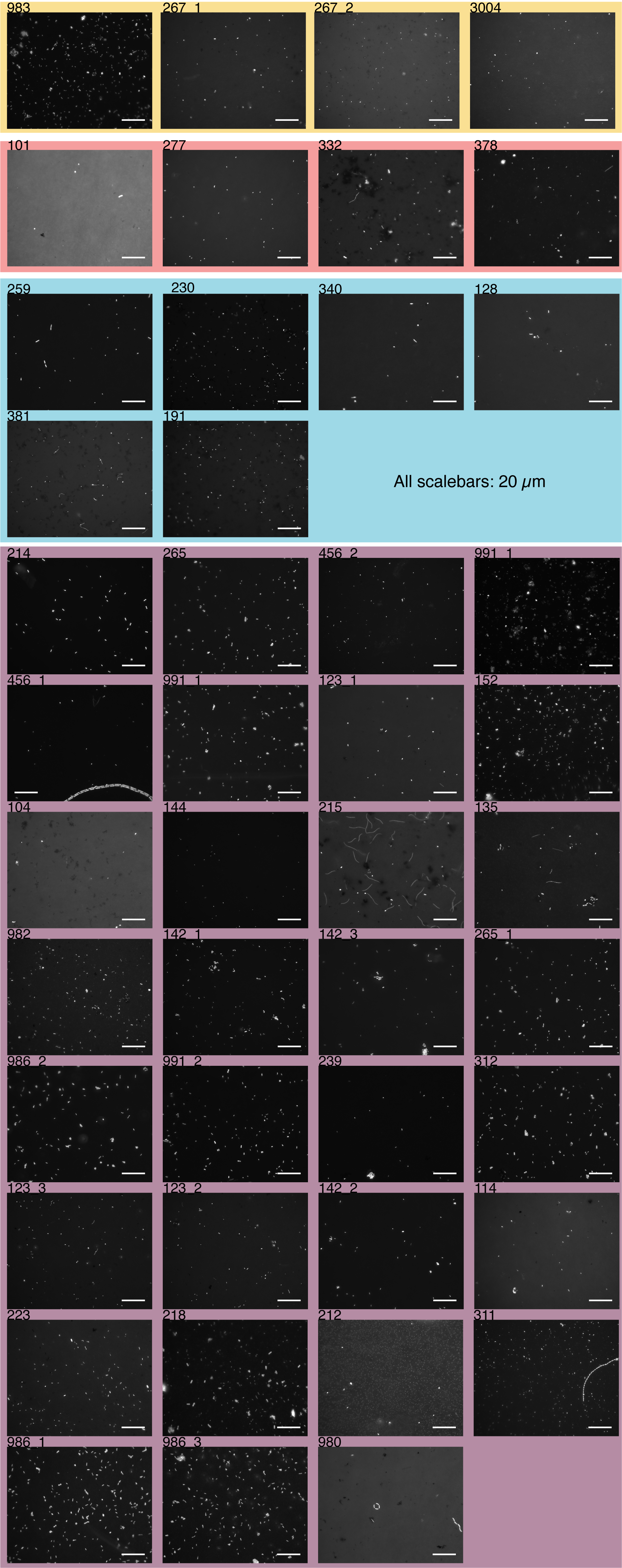

Supplement: Supplementary file 17 — Supplementary Dataset 14 [file 41467_2023_38523_MOESM17_ESM.zip › Raw_Data/Supplementary_Fig3_Ruff_et_al_Micrographs.tif]
