# Supplementary material for: Hydrogen and dark oxygen drive microbial productivity in diverse groundwater ecosystems
Source: Nat Commun. 2023 Jun 13;14:3194. doi: 10.1038/s41467-023-38523-4 (PMC10264387; doi:10.1038/s41467-023-38523-4)
Supplement: Supplementary file 18 — Reporting Summary [file 41467_2023_38523_MOESM18_ESM.pdf]

nature portfolio

Corresponding author(s): S. Emil Ruff

Last updated by author(s): Apr 27, 2023

Reporting Summary

Nature Portfolio wishes to improve the reproducibility of the work that we publish. This form provides structure for consistency and transparency in reporting. For further information on Nature Portfolio policies, see our Editorial Policies and the Editorial Policy Checklist.

Statistics

For all statistical analyses, confirm that the following items are present in the figure legend, table legend, main text, or Methods section.

n/a	Confirmed	
☐	☒	The exact sample size (*n*) for each experimental group/condition, given as a discrete number and unit of measurement
☐	☒	A statement on whether measurements were taken from distinct samples or whether the same sample was measured repeatedly
☐	☒	The statistical test(s) used AND whether they are one- or two-sided<br>*Only common tests should be described solely by name; describe more complex techniques in the Methods section.*
☐	☒	A description of all covariates tested
☐	☒	A description of any assumptions or corrections, such as tests of normality and adjustment for multiple comparisons
☐	☒	A full description of the statistical parameters including central tendency (e.g. means) or other basic estimates (e.g. regression coefficient) AND variation (e.g. standard deviation) or associated estimates of uncertainty (e.g. confidence intervals)
☐	☒	For null hypothesis testing, the test statistic (e.g. *F*, *t*, *r*) with confidence intervals, effect sizes, degrees of freedom and *P* value noted<br>*Give P values as exact values whenever suitable.*
☒	☐	For Bayesian analysis, information on the choice of priors and Markov chain Monte Carlo settings
☒	☐	For hierarchical and complex designs, identification of the appropriate level for tests and full reporting of outcomes
☒	☐	Estimates of effect sizes (e.g. Cohen's *d*, Pearson's *r*), indicating how they were calculated

Our web collection on statistics for biologists contains articles on many of the points above.

Software and code

Policy information about availability of computer code

Data collection	To generate and process chromatography, mass spectrometry, isotope-ratio mass spectrometry, microscopy, and gene/genome sequencing data we used the software of the instruments as provided by the instrument manufacturers. The instruments that we used are included in the materials section.
Data analysis	Raw amplicon sequences were analyzed using DADA2 v1.16. Amplicon-based community analyses were done using the custom workflow VisuaR v02 available on GiHub (https://github.com/EmilRuff/VisuaR). VisuaR is based on the software environment R v4.1.0 and features the following packages:<br>tibble_3.1.2 RColorBrewer_1.1-2 openxlsx_4.2.4 indicspecies_1.7.9 venn_1.10 UpSetR_1.4.0<br>ape_5.5 EnvStats_2.4.0 ggsignif_0.6.2 reshape2_1.4.4 plotrix_3.8-1 stringr_1.4.0<br>ggpubr_0.4.0 ggplot2_3.3.5 plyr_1.8.6 vegan_2.5-7 lattice_0.20-44 permute_0.9-5<br>cowplot_1.1.1 scales_1.1.1 tidyselect_1.1.1 purrr_0.3.4 splines_4.1.0 carData_3.0-5 colorspace_2.0-2 vctrs_0.3.8<br>generics_0.1.0 mgcv_1.8-36 utf8_1.2.1 rlang_0.4.11 pillar_1.6.1 glue_1.4.2 withr_2.4.2 DBI_1.1.1<br>lifecycle_1.0.0 munsell_0.5.0 gtable_0.3.0 zip_2.2.0 labeling_0.4.2 parallel_4.1.0 fansi_0.5.0<br>broom_0.8.0 Rcpp_1.0.6 admisc_0.16 polynom_1.4-0 backports_1.2.1 abind_1.4-5 farver_2.1.0<br>gridExtra_2.3 digest_0.6.27 stringi_1.6.2 rstatix_0.7.0 dplyr_1.0.7 grid_4.1.0 tools_4.1.0<br>magrittr_2.0.1 cluster_2.1.2 crayon_1.5.1 tidyr_1.1.3 car_3.0-13 pkgconfig_2.0.3 MASS_7.3-54<br>ellipsis_0.3.2 Matrix_1.3-4 assertthat_0.2.1 R6_2.5.0 nlme_3.1-152 compiler_4.1.0<br>Metagenome sequence datasets were processed and analyzed using BBDuk and BBMap both v38.90, reads were assembled using Megahit v1.2.9, contigs were binned using MetaBat2 v2.15, CONCOCT v1.1.0 and MaxBin2 v2.2.7. DAS-Tool v1.1.3 was used to integrate MAGs produced by the three binning tools. The contamination and completeness of MAGs were assessed by CheckM v1.2.0. MAG classification

using GTDB-tk (version 2.1.0, database release r207). Transfer RNA, ribosomal RNA, CRISPR elements, and protein-coding genes including nitric oxide dismutase coding genes and perchlorate dismutase coding genes were predicted and annotated using MetaErg v2.2.x. Full-length 16S/18S rRNA gene sequences were reconstructed from metagenomes using phyloFlash v3.4 and compared to ASVs using blastn v2.12.0. Amino acid sequences were aligned using Clustal Omega v1.2.4.

For manuscripts utilizing custom algorithms or software that are central to the research but not yet described in published literature, software must be made available to editors and reviewers. We strongly encourage code deposition in a community repository (e.g. GitHub). See the Nature Portfolio guidelines for submitting code & software for further information.

Data

Policy information about availability of data

All manuscripts must include a data availability statement. This statement should provide the following information, where applicable:
- Accession codes, unique identifiers, or web links for publicly available datasets
- A description of any restrictions on data availability
- For clinical datasets or third party data, please ensure that the statement adheres to our policy

The archaeal and bacterial 16S rRNA amplicon data generated in this study have been deposited in the NCBI SRA archive under BioProject accession number PRJNA861683 (https://www.ncbi.nlm.nih.gov/bioproject/PRJNA861683). The shotgun metagenomic data and metagenome-assembled genomes (MAGs) have been deposited in the SRA archive under BioProject accession number PRJNA700657 (https://www.ncbi.nlm.nih.gov/bioproject/PRJNA700657). The comprehensive environmental data generated in this study have been deposited in the PANGAEA archive under accession number 952473 (https://doi.pangaea.de/10.1594/PANGAEA.952473).

Human research participants

Policy information about studies involving human research participants and Sex and Gender in Research.

Reporting on sex and gender	NA
Population characteristics	NA
Recruitment	NA
Ethics oversight	NA

Note that full information on the approval of the study protocol must also be provided in the manuscript.

Field-specific reporting

Please select the one below that is the best fit for your research. If you are not sure, read the appropriate sections before making your selection.

☐ Life sciences ☐ Behavioural & social sciences ☒ Ecological, evolutionary & environmental sciences

For a reference copy of the document with all sections, see nature.com/documents/nr-reporting-summary-flat.pdf

Ecological, evolutionary & environmental sciences study design

All studies must disclose on these points even when the disclosure is negative.

Study description	This study is a survey of the microbiology and biogeochemistry of groundwater aquifers across a large geographical and geological scale in the Canadian Prairie. It includes >100 sampled groundwater wells, and at each well we carried out at least 50 physicochemical and microbiological analyses
Research sample	We collected water samples, gas samples, biomass samples at all monitoring wells
Sampling strategy	To ensure comprehensive and reliable insights into groundwater ecosystems we have collected environmental samples spanning a large geographic range (~210,000 square kilometers, about the size of England), a range of geological settings (14 major aquifers), a range of aquifer depths (5-230 m), and a range of years (2016-2020).
Data collection	The groundwater samples were collected from wells that are part of the Groundwater Observation Well Network which is run by the ministry of Environment and Parks, Alberta. Sample collection is carried out by a professional team dedicated to monitor groundwater quality in the Canadian province of Alberta
Timing and spatial scale	Samples were taken between 2016 and 2020. The total study area was ~210,000 square kilometers.
Data exclusions	We excluded sequencing datasets that had very low numbers of reads and can thus be considered failed runs, despite the generally low biomass in our samples. The threshold we used were 2000 reads (bacteria) and 1000 reads (archaea).
Reproducibility	We have included biological replicates (samples from the same well and timepoint), aquifer replicates (samples from the same well,

but from different years), technical replicates (samples that were split into subsamples and processed), as well as contamination controls (e.g., samples that were stored in the fridge and were samples several times across many weeks). We have used methods that corroborate each other (e.g., age dating was done using 14C and 3H) and we have physicochemical and biogeochemical data time series for many of the studied wells. Some of these wells have been studied every other year for several decades. The observed trends were very similar, if not identical, in all these samples and methods.

Randomization	NA
Blinding	We have included samples that were replicates of groundwater wells, but had different names. The correct wells/names were revealed after data processing.

Did the study involve field work? ☒ Yes ☐ No

Field work, collection and transport

Field conditions	Field work was carried out every year (2016-2020) between May and October. Weather conditions may have differed considerably, but the field gear and sampling protocols were standardized.
Location	Southern Alberta, Canada
Access & import/export	NA
Disturbance	No new monitoring wells were drilled for this study. The accessed wells were used for monitoring for many years to decades and the environmental disturbance is minimal.

Reporting for specific materials, systems and methods

We require information from authors about some types of materials, experimental systems and methods used in many studies. Here, indicate whether each material, system or method listed is relevant to your study. If you are not sure if a list item applies to your research, read the appropriate section before selecting a response.

Materials & experimental systems

n/a	Involved in the study
☒	☐ Antibodies
☒	☐ Eukaryotic cell lines
☒	☐ Palaeontology and archaeology
☒	☐ Animals and other organisms
☒	☐ Clinical data
☒	☐ Dual use research of concern

Methods

n/a	Involved in the study
☒	☐ ChIP-seq
☒	☐ Flow cytometry
☒	☐ MRI-based neuroimaging
